# Evidence of a Sjögren’s disease–like phenotype following COVID-19 in mice and humans

**DOI:** 10.1172/jci.insight.166540

**Published:** 2023-12-22

**Authors:** Yiran Shen, Alexandria Voigt, Laura Goranova, Mehdi Abed, David E. Kleiner, Jose O. Maldonado, Margaret Beach, Eileen Pelayo, John A. Chiorini, William F. Craft, David A. Ostrov, Vijay Ramiya, Sukesh Sukumaran, Ashley N. Brown, Kaley C. Hanrahan, Apichai Tuanyok, Blake M. Warner, Cuong Q. Nguyen

**Affiliations:** 1 Department of Infectious Diseases and Immunology, College of Veterinary Medicine, University of Florida, Gainesville, Florida, USA.; 2 Salivary Disorder Unit, National Institute of Dental and Craniofacial Research, NIH, Bethesda, Maryland, USA.; 3 Laboratory of Pathology, Center for Cancer Research, National Cancer Institute, NIH, Bethesda, Maryland, USA.; 4 AAV Biology Section, National Institute of Dental and Craniofacial Research, NIH, Bethesda, Maryland, USA.; 5 Center for Oral Health Integration, HealthPartners Institute, Bloomington, MN, USA.; 6 Department of Comparative, Diagnostic, and Population Medicine, College of Veterinary Medicine, and; 7 Department of Pathology, Immunology & Laboratory Medicine, College of Medicine, University of Florida, Gainesville, Florida, USA.; 8 LifeSouth Community Blood Centers, Gainesville, Florida, USA.; 9 Valley Children’s Hospital, Madera, California.; 10 Institute for Therapeutic Innovation, Department of Medicine, University of Florida College of Medicine, Orlando, Florida, USA.; 11 Department of Oral Biology, College of Dentistry and; 12 Center of Orphaned Autoimmune Diseases, University of Florida, Gainesville, Florida, USA.

**Keywords:** Autoimmunity, COVID-19, Rheumatology

## Abstract

Sjögren’s Disease (SjD) is a systemic autoimmune disease characterized by lymphocytic inflammation of the lacrimal and salivary glands (SG), dry eyes and mouth, and systemic symptoms. SARS-CoV-2 may trigger the development or progression of autoimmune diseases. To test this, we used a mouse model of SARS-CoV-2 infection and convalescent patients’ blood and SG in order to understand the development of SjD-like autoimmunity after infection. First, SARS-CoV-2–infected human angiotensin-converting enzyme 2 (ACE2) transgenic mice exhibited decreased salivation, elevated antinuclear antibodies (ANA), and lymphocytic infiltration in the lacrimal and SG. The sera from patients with COVID-19 sera showed increased ANA (i.e., anti-SSA [Sjögren’s-syndrome-related antigen A]/anti-Ro52 and anti-SSB [SS-antigen B]/anti-La). Male patients showed elevated anti-SSA compared with female patients, and female patients exhibited diverse ANA patterns. SG biopsies from convalescent COVID-19 patients were microscopically similar to SjD SG with focal lymphocytic infiltrates in 4 of 6 patients and 2 of 6 patients exhibiting focus scores of at least 2. Lastly, monoclonal antibodies produced in recovered patients blocked ACE2/spike interaction and cross-reacted with nuclear antigens. Our study shows a direct association between SARS-CoV-2 and SjD. Hallmark features of SjD-affected SGs were histologically indistinguishable from convalescent COVID-19 patients. The results implicate that SARS-CoV-2 could be an environmental trigger for SjD.

## Introduction

Sjögren’s Disease (SjD) is an autoimmune disease generally categorized by sicca symptoms in the mouth and eyes, autoantibodies, and lymphocytic infiltration into the salivary gland (SG) (1, 2). It is estimated that approximately 4 million Americans are affected, making SjD the second most common autoimmune disease after rheumatoid arthritis (RA) (3–5). SjD has the most skewed sex distribution (9:1 ratio of women to men) when compared with RA, multiple sclerosis (MS), and myasthenia gravis (6). SjD is most closely associated with symptoms of dryness, particularly of the mouth and eyes; however, a wide variety of extraglandular manifestations have been reported involving virtually any organ or tissue (4, 7). The extraglandular manifestations of SjD have been subdivided into visceral (gastrointestinal tract, lungs, heart, central, and peripheral nervous system) and nonvisceral (muscles, joints, and skin) involvement, indicating the wide variety of tissues that may be involved in the disease. While both men and women at any age can be affected by SjD, it is most commonly diagnosed in women in the fourth or fifth decade of life (7, 8). The pathological framework of SjD pathogenesis remains elusive; however, studies have suggested the primary drivers are genetic susceptibility, hormonal factors, and environmental triggers.

In December 2019, a novel coronavirus, SARS-CoV-2, emerged in Wuhan, Hubei Province, China, initiating a breakout of atypical acute respiratory disease, termed COVID-19. SARS-CoV-2 is a *betacoronavirus* in the family of *Coronaviridae*; the virus contains 4 structural proteins: S (spike), E (envelope), M (membrane), and N (nucleocapsid); 16 nonstructural proteins (nsp1−16); and 11 accessory proteins, which support viral essential physiological function and evasion from the host immune system (9). As of May 1, 2022, approximately 1 million US residents had died from COVID-19 (10) with more than 80 million total cases. Recent studies have identified the association between SARS-CoV-2 infection and autoimmune response. In point of fact, a recent literature review (11) (*n* = 1,176 articles and 90 case reports) revealed that the primary rheumatic diseases associated with patients with COVID-19 were vasculitis, arthritis, idiopathic inflammatory myopathies, and systemic lupus erythematosus. Several studies have found an association between antinuclear antibodies (35.6%) and COVID-19 infection, where the leading reactive antigens include SSA/Ro (25%), rheumatoid factor (19%), lupus anticoagulant (11%), and type I IFN (IFN-I) (10%) (12–14). In 6 independent case studies, patients with COVID-19 were diagnosed with systemic sclerosis (15), adult-onset Still’s disease (16), sarcoidosis, and systemic lupus erythematosus (SLE), with 4 of 6 patients acutely manifesting during COVID-19. An elevated level of anti-SSA/Ro52 in patients with COVID-19 was linked to pneumonia severity and poor prognosis (17). The underlying mechanism for the production of autoantibodies in patients with COVID-19 is unknown. However, it poses a significant challenge for post–COVID-19 symptoms or postacute sequelae of SARS-CoV-2 (PASC).

There are, additionally, reports and cases of patients with COVID-19 experiencing ocular and oral symptoms. Keratoconjunctivitis was observed in a few patients during a specific phase of the disease (18). One study has shown that xerostomia was observed in 29% of the patient cohort (19), while another showed an increase of 30% in reporting xerostomia during hospitalization (20). While these early studies had small sample sizes, the results indicated an association between COVID-19 and oral and ocular manifestations, primary symptoms of SjD. Increased rates of xerostomia in this patient cohort may be explained by the tropism of SARS-CoV-2 in the SGs, resulting in host immune response and immune-mediated injury (21). Furthermore, growing evidence of autoantibody production in patients with COVID-19 raises a critical question of whether SARS-CoV-2 infection is a risk factor for primary SjD. Therefore, the goal of this study was to determine the autoimmune response triggered by SARS-CoV-2 infection. The results indicate that infection with SARS-CoV-2 recapitulated an SjD-like phenotype in transgenic mice, and patients exhibited lymphocytic sialadenitis. Furthermore, the sera of patients with COVID-19 showed an increased frequency of antinuclear autoantibodies and levels of anti-SSA/Ro52 and anti-SSB/La compared with healthy controls (HCs).

## Results

### SARS-CoV-2 triggered a decrease in the salivary secretory function.

**Figure 1:**
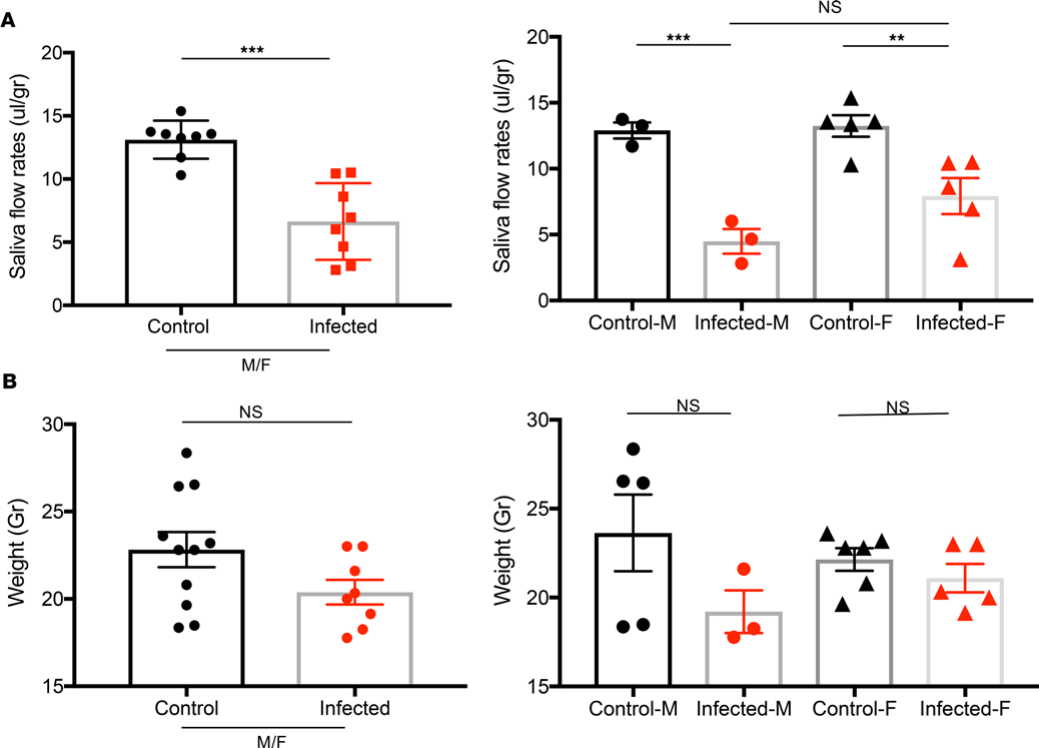
Decrease in saliva secretion by SGs by SARS-CoV-2. (**A**) Saliva flows were collected as described in the Methods section. The data shown represent the saliva flow rate (μL/gram). The mice were randomly selected for saliva collection at the endpoint (control/uninfected *n* = 8 and infected *n* = 8). To minimize the exposure of working in a BSL-3 mouse colony, a smaller number of mice was chosen for saliva collection. (**B**). The weight of the mice in grams (control *n* = 11, infected *n* = 8). Data were presented as mean ± SEM. One-tailed Mann-Whitney *U* tests were performed to determine significance, where **P < 0.01, ***P < 0.001.

Patients with SjD experience xerostomia, primarily due to the SGs’ diminished secretory function. In the spontaneous animal models of SjD, the secretory dysfunction occurs at 15–20 weeks of age. Here, we sought to determine if SARS-CoV-2 can compromise saliva secretion by the glands. The homozygous K18-hACE2 mice were i.n. inoculated with 860 PFU of SARS-CoV-2 WA1/2020 inoculum drop-by-drop into both nostrils until fully inhaled. Saliva was collected on day 21 prior to euthanasia. The infected male and female mice displayed a significant loss of salivary flow rates compared with the uninfected male and female mice (infected: 6.64 ± 1.075 vs. uninfected: 13.12 ± 0.532 μL/gr). The infected males appeared to lose more saliva flow than infected females; however, the loss of saliva between infected males and females was not statistically significant (Figure 1A). To eliminate the probability that the change in the salivary flow rate was due to the change in weight as opposed to the saliva production, the weight change was calculated as well. The infected mice showed a decrease in body weight compared with the uninfected mice; however, the decrease was not statistically significant (Figure 1B). The results suggest that SARS-CoV-2 infection has a negative effect on the secretory function of the SGs in both males and females.

### SARS-CoV-2 induced the production of autoantibodies.

**Figure 2:**
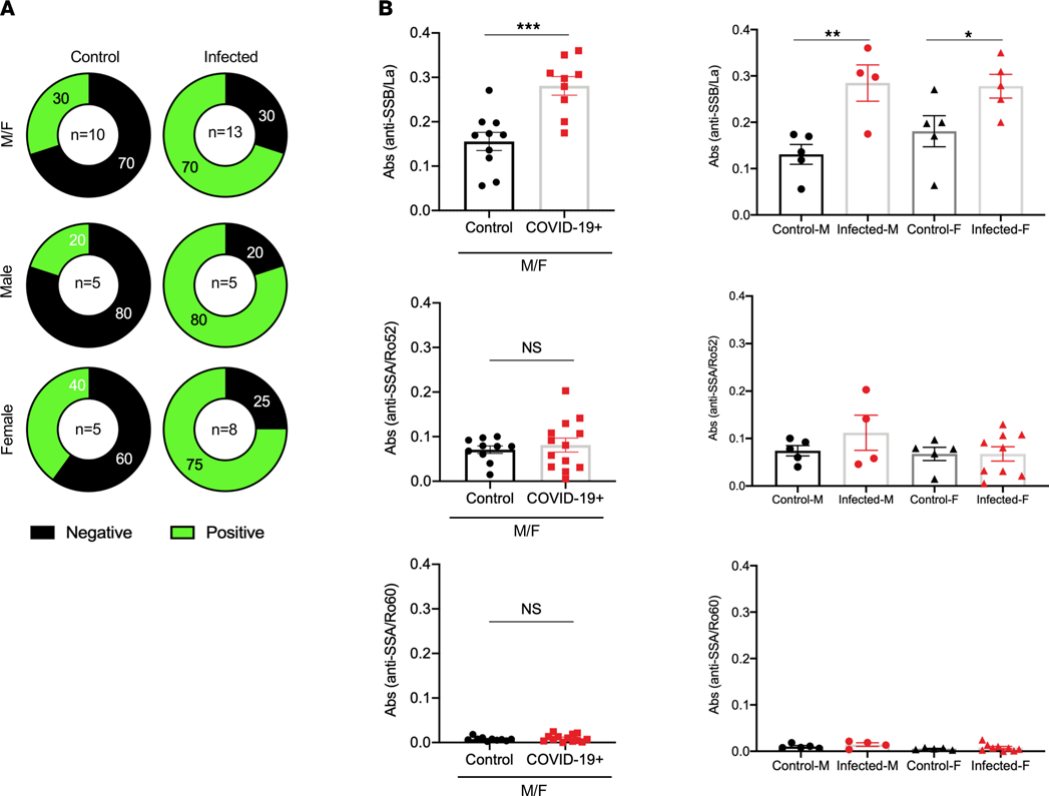
Autoantibody profile of mouse sera. (**A**) Antinuclear antibody profile was determined using HEp2 cells, where M/F indicates a combined antinuclear antibody profile, as opposed to male and female mice listed separately. A Chi-squared test was performed on the all control (*n* = 10, 5 females, 5 males) and SARS-CoV-2–infected mice (*n* = 13, 6 males, 7 females), with a value of 32, *P* < 0.00001; females: χ^2^ = 25.0639, *P* < 0.00001; and males: χ^2^ = 72; *P* < 0.00001. Sera were diluted at 1:40. (**B**) Anti-SSB/La, anti-SSA/Ro52, and anti-SSA/Ro60 were determined using ELISA. Welch’s *t* test was performed to determine significance, where *P = 0.026, **P = 0.01, and ***P = 0.0003. On the left, a combined profile is provided (M/F), and on the right, results are separated by sex. M, male; F, female.

Patients with SjD generally develop antinuclear antibodies, including anti-SSA/Ro. Here, we sought to determine if SARS-CoV-2 infection induced autoantibody production. As presented in Figure 2A, 70% of all infected mice were positive, and 30% were negative for antinuclear antibodies using HEp2 cell staining. In the inverse, of the uninfected mice, 70% of both sexes were negative, and 30% were positive for antinuclear antibodies. Interestingly, males increased from 20% positive for antinuclear antibodies to 80% positive following infection, and females only changed from 40% positive for antinuclear antibodies to 75% positive for antinuclear antibodies. Furthermore, we examined the SjD-associated autoantibodies. As indicated in Figure 2B, anti-SSB/La levels were highly elevated in the combined infected group compared with the control group. There was no difference in anti-SSA/Ro52 and anti-SSA/Ro60 levels between the control and infected groups. Similar patterns were observed when we compared the males and females separately. The results suggest that SARS-CoV-2 infection in mice promotes the development of antinuclear antibodies with higher frequency in males and specific autoantibodies associated with SjD.

### SARS-CoV-2 caused apoptosis and inflammation in the lacrimal glands and SGs of mice.

**Figure 3:**
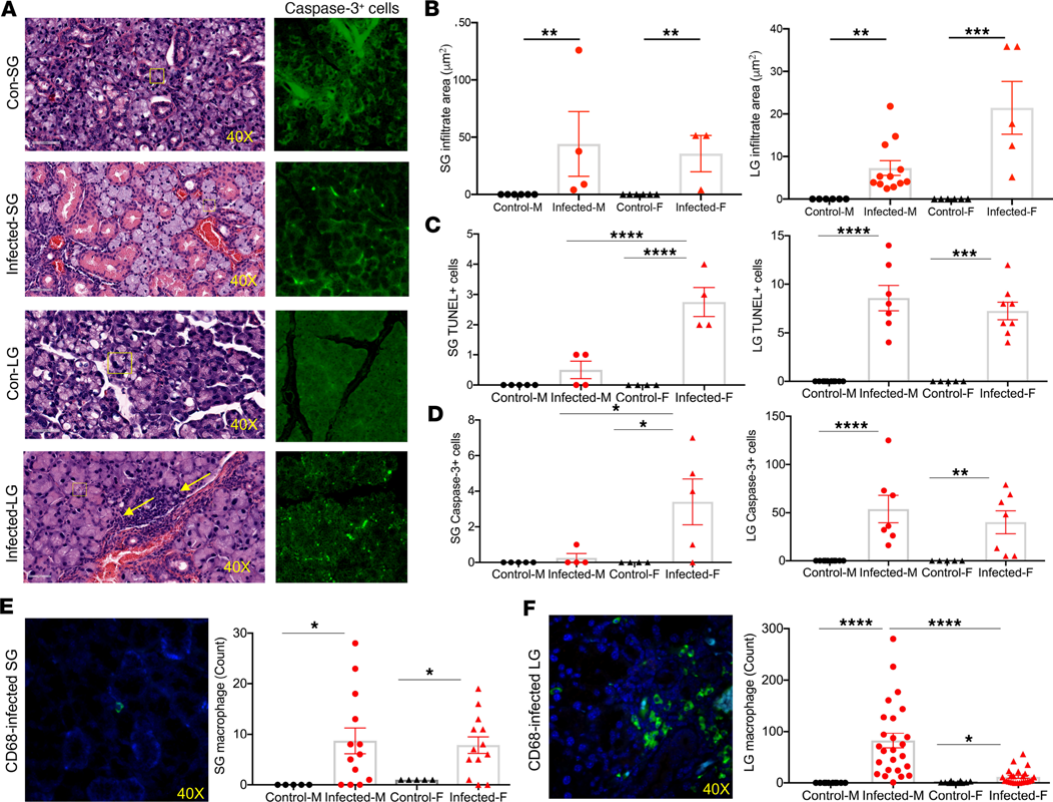
Increase in inflammation and apoptosis detected in SGs and LGs of infected mice. (**A**) Representative H&E staining of the SGs and LGs of the control and SARS-CoV-2–infected mice with caspase-3^+^ cells. Yellow arrows indicate lymphocytic infiltrates in the interstitium. (**B**) Larger areas of lymphocytic infiltration are present in the exocrine glands of infected mice. The lymphocytic focal areas of 52 LGs and 26 SGs were counted using Aperio ImageScope (v12.4.6.5003), with each point representing 1 countable focus in the SG or LG (control, *n* = 5 females; infected, *n* = 26, 13 males, 13 females). (**C**) Elevated glandular apoptosis detected by TUNEL staining (SGs: control males *n* = 5, control females *n* = 4, infected males *n* = 4, and infected females *n* = 5; LGs: control males *n* = 10, control females *n* = 5, infected males *n* = 7, and infected females *n* = 8). (**D**) Elevated glandular apoptosis detected by caspase-3 staining (SGs: control males *n* = 5, control females *n* = 4, infected males *n* = 4, and infected females *n* = 5; LGs: control males *n* = 10, control females *n* = 5, infected males *n* = 7, and infected females *n* = 8). (**E**) Increase in CD68^+^ macrophage frequency in SGs and (**F**) LGs of the infected mice. Representative immunofluorescence staining of CD68^+^ macrophages are displayed in green with blue DAPI nuclei staining. Original magnification, ×40. The statistical significance was calculated using 1-tailed Mann-Whitney *U* tests where error bars indicate SEM; *P < 0.05, **P < 0.01, ***P < 0.001, and ****P < 0.0001. M, male; F, female.

The principal targeted tissues for SjD are the lacrimal glands (LGs) and SGs. The inflammatory lesions are composed of a multitude of immune cell types, notably B cells, T cells, and macrophages. As presented in Figure 3A, the LGs of infected mice had multifocal apoptosis by caspase-3 staining of low to moderate numbers of acinar epithelial cells characterized by cells with condensed, hypereosinophilic cytoplasm and pyknotic nuclei with karyorrhexis. The apoptosis/necrosis resulted in variable collapse and loss of acini. The interlobular duct epithelium was unaffected. The SGs and LGs of infected mice occasionally had small interstitial lymphocytic infiltrates, characteristic of immune stimulation and response. SG and LG interstitial lymphocytes were not present in the noninfected mice. Examining the infiltrate areas revealed that both males and females showed an increase in the LGs and SGs, with more severe lacrimal infiltrates in the infected females than in males (Figure 3B). We further examined the apoptotic levels using TUNEL and caspase-3. As indicated in Figure 3, C and D, infected female mice showed a significant increase in TUNEL^+^ and caspase-3^+^ cells in both glands. Interestingly, infected females exhibited higher levels of salivary TUNEL^+^ cells compared with infected male mice.

To further quantify the lymphocytes as a response to inflammation, we performed staining for CD3^+^ T cells, B220^+^ B cells, and CD68^+^ macrophages (Figure 3, E and F; Supplemental Figure 1, A and B; supplemental material available online with this article; https://doi.org/10.1172/jci.insight.166540DS1). A few infected female mice showed increased CD3^+^ T cells and B220^+^ B cells in the SGs; however, both sexes showed a substantial uptick in macrophages. In contrast, only infected males showed an increase in CD3^+^ T cells and B220^+^ B cells in the LGs, with a significant increase of macrophages in both males and females. Remarkably, infected males showed elevated lacrimal macrophage populations compared with infected females (Figure 3F). Lastly, we correlated these data with the presence of lymphocytic foci. Only a single infected mouse developed a focus score (FS) in the SGs, so deviation from the control group is insignificant (χ^2^ =.27, *P* = 0.10247). However, FSs were detected in the LGs of 5 infected mice (χ^2^ = 13, *P* = 0.00031) (Supplemental Table 1). Additionally, lymphocytic infiltration of B and/or T cells that do not qualify as a focus were examined. This indicates localized inflammation in the SGs (χ^2^ = 11, *P* = 0.00091) and LGs (χ^2^ = 24, *P* = 0.00001). Overall, SARS-CoV-2 induced inflammation with multifocal apoptosis with more severity in the LGs than SGs.

### COVID-19 is associated with higher autoantibody levels in a sex-specific manner.

**Figure 4:**
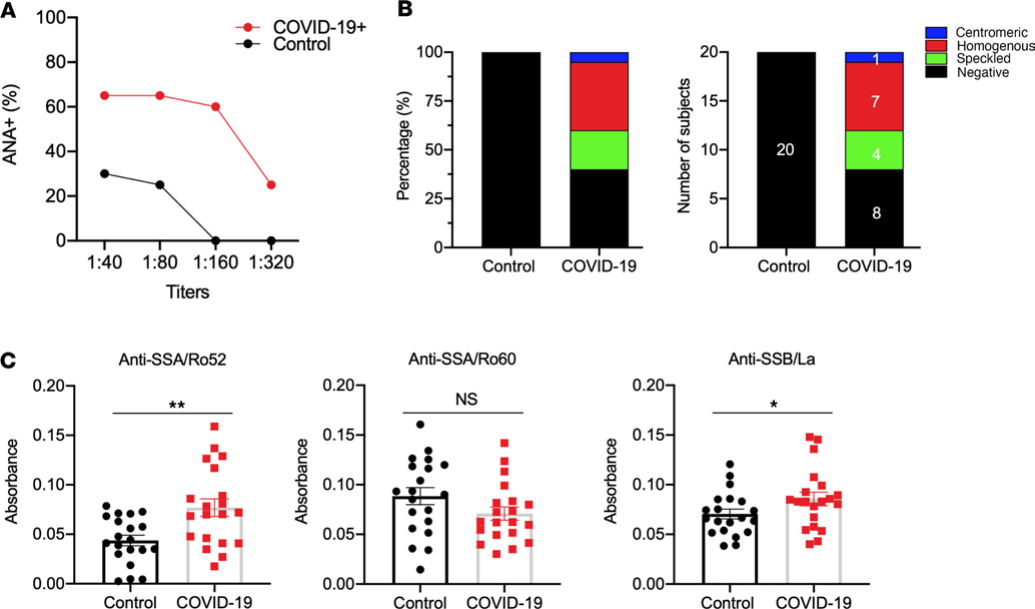
Autoantibody induction in COVID-19 human sera. (**A**) ANA profile was determined using HEp2 slides at various sera titers (*n* = 20 patients with COVID-19^+^ comprising 10 males and 10 females, *n* = 20 HCs with 10 males and 10 females). (**B**) A breakdown by specific ANA staining pattern at 1/320 serum titer is presented showing the percentages (left) and number (right) of patients. (**C**) Anti-SSB/La, anti-SSA/Ro52, and anti-SSA/Ro60 were determined using ELISA. Welch’s *t* test was performed to determine significance, where *P = 0.0415, **P = 0.0015. ANA, antinuclear antibodies.

As described above, mice infected with SARS-CoV-2 developed antinuclear antibodies and elevated anti-SSB/La. Here, we sought to determine if these findings were also observed in human patients. As presented in Figure 4A, the patients with COVID-19 exhibited higher frequencies of positive antinuclear antibodies at different sera titers compared with HCs. Notably, 60% of patients showed positive antinuclear antibodies, with none for HCs at 1:160 titer, and 30% of patients still exhibiting positive antinuclear antibodies at 1:320 titer. Further analysis of the staining patterns revealed that among the positive antinuclear antibodies for patients, 40% (7) were homogeneous, 15% (4) were speckled, and 5% (1) were centromeric (Figure 4B). To further determine if the patients with COVID-19 presented with SjD signature autoantibodies, patient sera were examined for reactivity against SSA/Ro52, SSA/Ro60, and SSB/La. As presented in Figure 4C, anti-SSA/Ro52 and anti-SSB/La were significantly elevated in patients with COVID-19 compared with HCs. Anti-SSB/Ro60 levels remained similar between the 2 groups.

SjD has a strong predilection for females; therefore, we sought to determine whether patients with COVID-19 exhibited an element of sexual dimorphism in the autoantibody response. Interestingly, when examining the antinuclear antibody staining, it was discovered that the female patients with COVID-19 had a significantly higher percentage of positive antinuclear antibodies at various titers than the male patients with COVID-19 or either sex of control patients (Supplemental Figure 2A). Additionally, the female patients with COVID-19 were shown to present a more diverse antinuclear antibody pattern, with 30% speckled, 40% homogenous, and 10% centromeric at 1:160 titer, whereas the male patients showed a 10% speckled and 30% homogenous pattern at the same titer. The female patients still exhibited 20% speckled, with males showing 10% speckled at 1:320 titer, and the homogenous pattern was 10% for both sexes. Positive staining for both sexes of the control group only contained a homogenous pattern, but were unsustained past 1:80 titer (Supplemental Figure 2B). To further determine if male and female patients with COVID-19 exhibited different levels of SjD-associated autoantibodies, we performed ELISAs on the sera based on sex. As presented in Supplemental Figure 2C, female and male patients with COVID-19 showed significantly higher levels of anti-SSA/Ro52 than their counterparts. Interestingly, male patients with COVID-19 showed elevated levels of anti-SSA/Ro52 above female patients with COVID-19 (*P* = 0.0029). There was no statistically significant difference between male and female patients with COVID-19 with anti-SSA/Ro60 or anti-SSB/La. The results indicated that female patients manifested more diverse patterns of antinuclear antibodies; however, male patients exhibited higher levels of anti-SSA/Ro52 than female patients.

### Monoclonal antibodies produced by patients with COVID-19 are reactive against nuclear antigens.

It is remarkable to observe the crossreactivity of the sera of patients with COVID-19 against self-antigens, as demonstrated here. To further evaluate the B cell response of patients with COVID-19, we produced and selected 9 monoclonal antibodies from convalescent COVID-19 patients by isolating CD20^+^ memory B cells reactive against both the receptor-binding domain (RBD) and S1 of SARS-CoV-2 and examined their reactivity against self-antigens. As presented in Supplemental Figure 3A, the monoclonal antibodies exhibited various degrees of inhibition against SARS-CoV-2 RBD, in which monoclonal antibodies A10 and B5 showed the highest inhibitory activity at varying dilutions using pseudovirus. To further support the infection inhibition capacity of the same monoclonal antibodies, we performed a Plaque Reduction Neutralization Test (PRNT) with SARS-CoV-2 USA-WA1/2020. We found that C9, A10, B5, and C7 showed high inhibition activity, especially at higher concentrations, compared with the negative control (Supplemental Figure 3B). Lastly, we tested them against HEp2 cells to determine their reactivity against nuclear antigens. As described in Supplemental Figure 3C, 7 of the 9 S1/RBD-reactive monoclonal antibodies produced a strong homogenous staining pattern at 1:40 and 1:80 titers and lowered to 67% (6 out of 9) at 1:160 and 1:320 titers. Examining the CDR3 sequences indicated that most of the monoclonal antibodies share 1 or 2 serine and glycine in the heavy chain and 1 tyrosine in the light chain. Clones A10 and B5 displayed the longest CDR3 regions (20aa and 23aa, respectively) in the heavy chain (Supplemental Table 2). Overall, the results demonstrate that monoclonal antibodies against the virus produced in patients who have recovered from COVID-19 are crossreactive and capable of recognizing nuclear antigens.

### Convalescent COVID-19 patients demonstrate inflammation of the SGs and clinical signs and symptoms of SjD.

**Figure 5:**
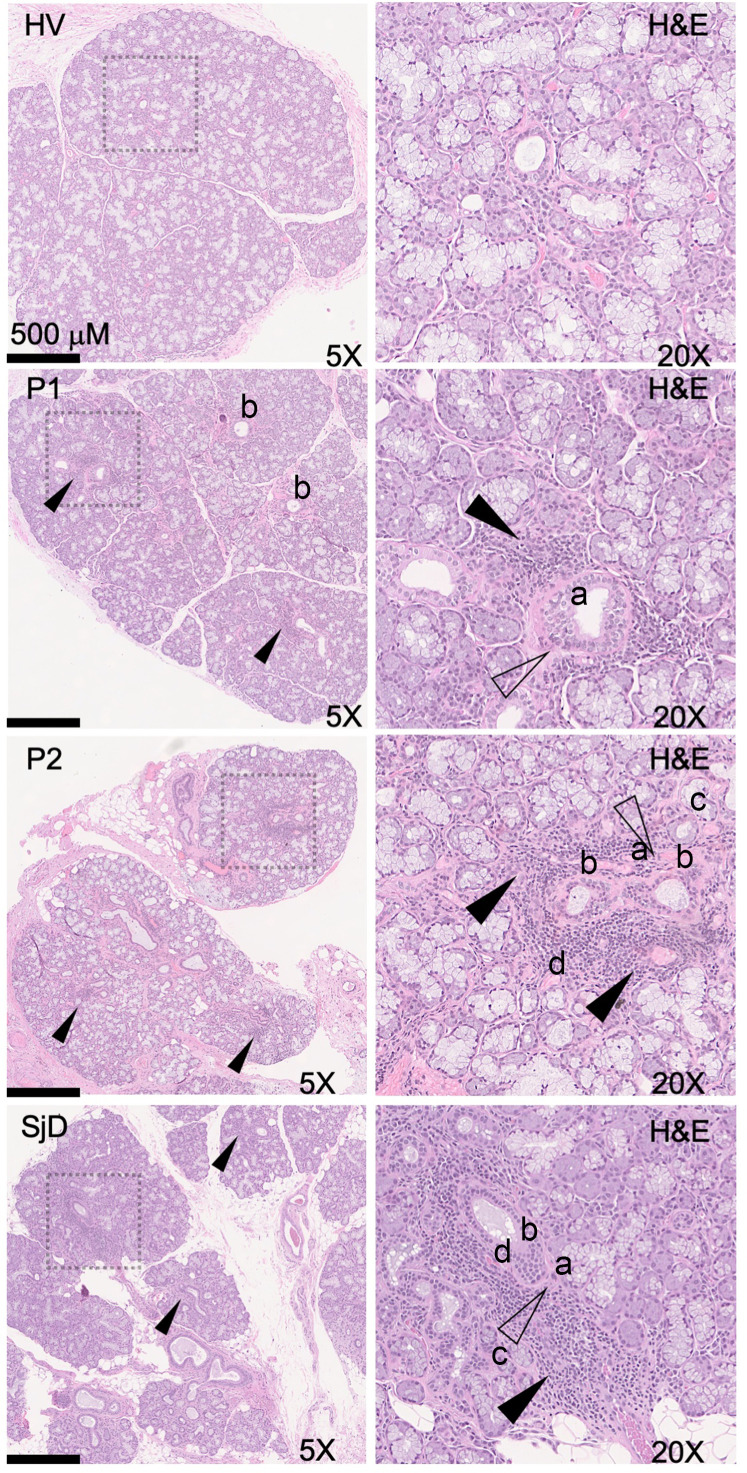
Representative MSG H&E photomicrographs of HCs, SjD, and 2 representative patients recovered from COVID-19. Convalescent glands exhibit a range of inflammation severity ranging from normal to mild-to-moderate sialadenitis with FLS reminiscent of inflammation found in SjD. The histopathological findings from 2 patients (P2 and P5) exhibit inflammation consistent with findings observed in SjD SGs (e.g., FLS with FSs > 1.0). However, P1, P3, and P6 exhibited FLS but did not reach the threshold of greater than 1.0 focus per 4 mm^2^ of tissue. Black arrowheads point to foci of inflammation, outlined arrowheads point to areas of fibrosis: a, ductal injury; b, mucous inspissation; c, immune infiltration of the acini with injury; and d, perivascular infiltrates. Total original magnification, 5× (left column), 20× (right column).

Six generally healthy, relatively young (Range: 19–42 yr; Mean: 31 yr) patients who had recovered from COVID-19 and had convalescent minor SG (MSG) biopsies were identified for this study. These patients were free from evidence of pre-existing autoimmune disease or major medical conditions. Patients 1–3 were enrolled on NIH IRB Protocol: 20-D-0094 and had convalescent MSG biopsies 6–13 months after recovery from COVID-19 (Table 1). In addition, Patient 2 also received an initial biopsy during acute COVID-19 (Table 1 and Figure 5). These patients recovered from COVID-19 without continued postacute COVID-19 symptoms as primary clinical concerns. Patients 4–6 were enrolled on an NIH IRB Protocol: 15-D-0051 as HCs and did not present with clinical complaints of SjD or postacute COVID-19 syndrome. Their COVID-19 status was determined from patient interviews and serological studies. In patients with known COVID-19, their clinical course was generally mild; 3 patients reported lung involvement with shortness of breath without hospitalization, and 1 patient reported significant gastrointestinal involvement (mild-to-moderate COVID-19). A single patient, Patient 5, was unaware of their post–COVID-19 status and was considered asymptomatic. Evidence of infection included clinical reports of infection in 5 of 6 patients, clinical nasopharyngeal swab PCR for SARS-CoV-2 N1 and N2 genes in 4 patients (Patients 1–3 and 6); antinucleocapsid antibodies were positive in all 6 patients (*data not shown*). No patients were positive for antinuclear antibodies or anti-SSA/Ro antibodies. A single patient was low-titer positive for anti-SSB/La antibodies. Three of the 6 patients reported dry mouth during acute COVID-19, sustained temporarily after recovery (up to 3 weeks); and a single patient had objective evidence of dry mouth (Patient 2) during acute COVID-19. Interestingly, this patient did not produce saliva from the submandibular glands for about 3 of the 4 weeks of weekly followup after infection. Dry eye assessments for 3 patients could not be completed in the NIH COVID-19 Testing Facility. Two of the 3 three patients who presented through the NIH Dental Clinic had objective evidence of dry eye disease (Table 1) but did not have clinical complaints of dry eyes.

Overall, 7 MSG biopsies were collected from 5 patients, and a single patient had serial biopsies. One biopsy occurred during acute COVID-19 5 days after symptom debut, and the second took place 6 months after recovery. Generally, biopsies exhibited mild chronic sialadenitis (Table 1). However, 5 of the 7 biopsies (from 4 of the 6 patients) had multiple foci (> 50 lymphocytes) of inflammation (e.g., focal lymphocytic sialadenitis, FLS; Table 1; Figure 5; and Supplemental Figure 4). Most foci were small, although several glands exhibited multiple medium-sized and coalescing foci. Mild fibrosis and atrophy of the glands were seen in 3 patients (Patients 1–3). It is noteworthy that Patient 2’s followup biopsy exhibited an increased FS (FS:1 → FS:2) and the elaboration of fibrosis and atrophy of the glands (Figure 5). Histopathological evidence of injury included ductal injury and mucous inspissation, immune infiltration of the acini with injury, perivascular infiltrates, and granuloma (examples illustrated in Figure 5 and Supplemental Figure 4). In some patients, the histopathological features in 4 of 6 patients (5 biopsies) are reminiscent of the range of histopathological features found in the MSG of patients with SjD.

**Figure 6:**
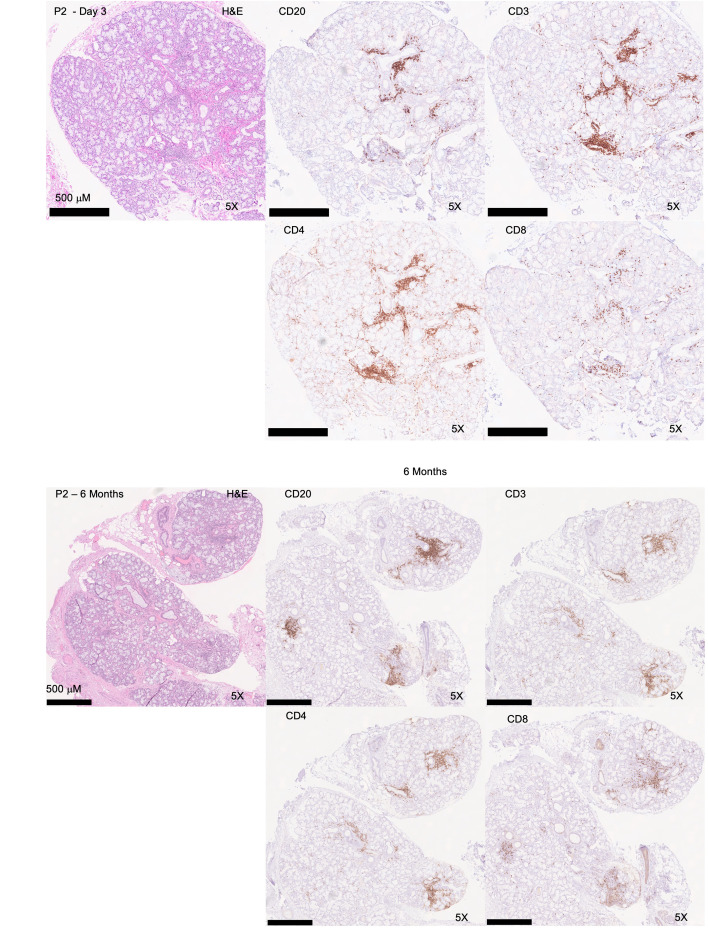
Immunophenotyping of acute and postacute COVID-19 infection. Representative immunophenotyping studies examining CD3, CD4, CD8, and CD20 on an MSG biopsy during infection (D5 after first symptom; FS: 1) and post–COVID-19 infection (6 months) (P2). Immunophenotyping demonstrates diffuse mild-to-moderate chronic sialadenitis with FLS. Original magnification, ×5.

To understand the composition of the immune infiltrates, clinical immunophenotyping was performed on 4 biopsies from 3 patients. The infiltrates are generally composed of varying proportions of T and B cells, with small foci predominantly composed of T cells and larger foci exhibiting a shifted balance toward B cell predominance. CD8 T cells were found scattered throughout the gland and in the inflammatory foci (Figure 6 and Table 2). These IHC studies are highly similar to the inflammatory infiltrates found characteristically in SjD. In the single patient (Patient 2) with followup MSG, the amount of inflammation and the shift to B cell predominance can be appreciated in the areas of FLS at 6 months.

## Discussion

Increasing evidence has supported the associations between viral/bacterial infections and autoimmune diseases. An early study demonstrated that murine cytomegalovirus induced an SjD-like disease in C57Bl/6-lpr/lpr mice with sialadenitis, severe SG inflammation, and production of anti-SSA/Ro and anti-SSB/La (22). Recent studies suggested that SARS-CoV-2 has a tropism for the SG, including SARS-CoV-2 (21, 23). Here, we sought to determine if SARS-CoV-2 infection could also trigger SjD-like phenotypes in a murine model. The results indicate that SARS-CoV-2 infection recapitulates several signature disease phenotypes, specifically, diminished salivary flow rates, SG and LG inflammatory lesions, and elevated autoantibodies. Similar findings were also observed in patients with COVID-19, in which significantly elevated levels of anti-SSA/Ro52 and anti-SSB/La were seen. Additionally, female patients manifested more diverse patterns of antinuclear antibodies, and male patients exhibited higher levels of anti-SSA/Ro52 than female patients. In summary, the data suggest that SARS-CoV-2 infection triggered an SjD-like disease in a murine model and in human patients.

SARS-CoV-2 primarily uses ACE2 as a receptor (24, 25), broadly expressed by endothelial and epithelial cells, including those of the aerodigestive tract and the SGs (21, 26–28). It has now been shown that SGs can robustly support infection and replication of SARS-CoV-2 and that saliva is potentially infectious and transmissible (21). Intraindividual spread of SARS-CoV-2 initiates from the epithelial cells of the upper respiratory tract (e.g., acinar and ductal cells of the SGs) by active replication and egress of offspring viruses subsequently infecting ACE2-expressing cells in downstream organs, including the heart, kidneys, gastrointestinal tract, and vasculature (21, 29, 30). The hACE2 transgenic model expressed high levels of hACE2 in the LGs and a lesser amount in the SGs (Supplemental Figure 5). Furthermore, LGs exhibited an elevated frequency of nucleocapsid-positive cells than the SGs (Supplemental Figure 6). Therefore, higher expression of hACE2 and nucleocapsid-positive cells could explain the more severe LG inflammation and cell death due to higher viral infection and replication. The viral loads in the lungs varied among the tested mice; however, the clinical features were not affected (Supplemental Figure 7). The kidneys and brains were examined and no abnormal pathology was noted (data not shown). ACE2 is expressed in squamous epithelial cells of the dorsal tongue, gingiva, and buccal tissue, and transmembrane serine protease 2 (TMPRSS2) is expressed in taste bud cells and submandibular glands (21). SARS-CoV-2 was detected in SGs, with higher levels in the MSGs (21). In addition, saliva is a natural reservoir for viruses as one of the major fluids for viral detection (31). Therefore, it is not surprising that SARS-CoV-2 was found in the SGs and facilitated the inflammatory response.

The severity of COVID-19 is mediated by unregulated inflammation (32). During the later stage of the disease, immune-mediated damage leads to a progressive increase in inflammation (33). Additionally, patients with life-threatening pneumonia had neutralizing autoantibodies against IFN-ω and IFN-α (14). As demonstrated, mice infected with SARS-CoV-2 developed higher antinuclear antibodies and anti-SSB/La levels. Similarly, patients developed elevated levels of antinuclear antibodies, specifically anti-SSA/Ro52 and anti-SSB/La. To determine whether the presence of autoantibodies that are characteristic of other autoimmune diseases, we performed the INNO-LIA antinuclear antibody Update Test strips (FujireBio Diagnostics), which detect 13 nuclear antigens. None of the infected mice were positive for the nuclear antigens. SSA/Ro60 was positive in a single female control patient, whereas 4 patients with COVID-19^+^ (1 male and 3 females) were positive. Additionally, ribosomal P was solely positive in a few patients at 1:320 serum dilution (Supplemental Figure 8). A potential pitfall of the assay is that it is a qualitative multiparameter Western blot immunoassay with limited sensitivity (34). In a study analyzing the sera and plasma from 64 patients with COVID-19, approximately 25% of patients exhibited an autoantibody response on average 12.3 days after diagnosis, and the reactivity was primarily to nuclear antigens, including ribonucleoprotein (RNP) (*n* = 8), SSA, SSB, dsDNA, chromatin, or centromere (35). Chang el al. showed that autoantibodies are present in approximately 15% of HCs and 50% of patients with COVID-19 against commonly recognized antigens in an array of autoimmune disorders, including SSA/Ro52 (36). However, Burbelo et al. demonstrated that a considerable fraction of the autoantibody positivity in patients with severe COVID-19 may be related to receiving i.v. Igs (37). Thus, these results suggest that longitudinally sampled and controlled serosurveillence must be performed.

A meta-analysis revealed that the development of primary rheumatic diseases associated with patients with COVID-19 were vasculitis, arthritis, idiopathic inflammatory myopathies, and systemic lupus erythematosus; overall, the association between antinuclear antibodies and COVID-19 infection was 35.6%, and the reactive antigens were found at the following rates: SSA (25%), rheumatoid factor (19%), lupus anticoagulant (11%), and IFN-I (10%) (11). Autoantibody responses in patients with COVID-19 can be influenced by sex, with men exhibiting an autoantibody response after an infection defined as at least mildly symptomatic, whereas women were prone to produce this response following an asymptomatic infection; thus, autoantibody profiles are highly variable between the sexes and dependent on the disease severity (38). It is unknown how SARS-CoV-2 infection could induce a plethora of autoantibodies, specifically those hallmarks of autoimmune diseases. One hypothesis is that the tropism of SARS-CoV-2 to vulnerable cells triggers a robust immune response that damages infected cells leading to the presentation of antiviral protein-viral particle-antibody immune complexes to antigen-presenting cells in the interstitium. A study showed that heptapeptide sharing exists between SARS-CoV-2 spike glycoprotein and human proteins, indicating the molecular mimicry mechanism (39). However, the spike protein does not share any homology with SjD-specific autoantigens. We found only 20% positive antinuclear antibodies for female patient sera and 30% for male sera at 1:320 titer, as recommended by the American College of Rheumatology (ACR) criteria for SjD (40). Even with the small number of male and female patients and controls, the finding may have potential clinical implications in diagnostics and treatment. The crossreactivity of monoclonal antibodies and antinuclear antigens by HEp2 cells was revealing, especially in the CDR3 regions. Most highly crossreactive monoclonal antibodies shared 1 or 2 serine and glycine in the heavy chain and 1 tyrosine in the light chain, with some having long CDR3 regions in the heavy chain. Previous studies have shown that these aa tend to be selected within CDR regions after affinity maturation, for example, serine and glycine are small neutral aa that allow structural flexibility in the antigen-binding site. Additionally, the large side chain of tyrosine would allow it to actively interact with residues at the antigen interface by hydrogen bonds as well as hydrophobic and attractive electrostatic interactions with positively charged groups (41). While long CDR3 regions have not been directly implicated in autoimmune diseases or SjD, studies have shown that the CDR3 lengths were similar between autoreactive and nonautoreactive Ig genes in patients with RA (42) and polyreactive IgM (43). Theoretically, a more extended CDR3 region could provide more potential interaction sites, albeit with reduced affinity for the target antigen. Regardless, it is imperative to determine the underlying mechanism of autoantibody response triggered by SARS-CoV-2.

We, and others, have confirmed that SGs are exquisitely supportive of infection and replication of SARS-CoV-2, and saliva is an ideal secretion for inter- and intraindividual spread of de novo virus (21, 44). Because of the long-hypothesized connection between viral infection and initiating autoimmune diseases, we examined available clinical data and MSG biopsies from convalescent COVID-19 patients with mild-to-moderate COVID-19 infections. While no patients satisfied strict 2016 ACR/EULAR classification criteria, FLS or clinical signs and symptoms of SjD were found in most available patients (40). In select patients, the histopathological features of inflammation in the SGs are indistinguishable from SjD and, in the proper clinical context, would be supportive of the diagnosis.

The most prevalent and persistent oral symptoms associated with COVID-19 include taste dysfunction. Furthermore, dry mouth as a result of hypofunction is often overlooked in patients with COVID-19 and was identified as another highly prevalent (43%) oral manifestation of COVID-19 (45). A review of 12 studies, including patients diagnosed with SARS-CoV-2 infection from different countries with reported oral symptoms associated with COVID-19 infection, showed that xerostomia occurs in the early stages of COVID-19 with a prevalence ranging from 20% to 61.9% and can persist for at least 8 months after recovery (46). The percentage is higher in patients with mild symptoms, as a study in Israel showed 61.9% of 97 confirmed nonhospitalized patients reported xerostomia (47). This is consistent with our data showing a markedly diminished salivary secretion after SARS-CoV-2 infection in mice. The precise etiology of gland dysfunction requires further investigation. As demonstrated, the influx of inflammatory cells in the glands, concomitantly with the rapid increase of acinar cell apoptosis, may contribute to diminished gland function. We did not measure tear secretion, mainly to avoid further physical stress on the mice as a result of the drug side effect and handling.

In summary, our study underpins the pathogenic role of SARS-CoV-2 in SjD. SARS-CoV-2 induced gland inflammation leading to the loss of saliva in mice. It triggered the production of SjD-associated autoantibodies in mice and human patients. Further studies are needed to examine the pathoetiology of SARS-CoV-2 in SjD, specifically investigating the underlying mechanisms contributing to cellular damage and immunological response development. Long-term followup studies are necessary to assess the impact of COVID-19 and the different variants on the disease course and progression of SjD by determining if COVID-19 triggers flares or exacerbations of symptoms and investigating potential long-term complications. Future studies may focus on developing guidelines and recommendations for managing individuals with SjD and COVID-19. These guidelines may address risk assessment, preventive measures, and optimal strategies for managing both conditions concurrently.

## Methods

### Human samples

SARS-CoV-2–positive and HC sera were obtained from the The University of Florida, Clinical and Translational Science Institute, Biorepository at the University of Florida in compliance with IRBs 202001475 and 2020000781. A comprehensive clinical diagnosis of the 20 controls and 20 patients was presented in Supplemental Table 3. The presence of SARS-CoV-2 was confirmed by reverse transcriptase PCR (RT-PCR) for admittance into the CTSI Biorepository Bank. PBMCs from 5 postconvalescent COVID-19 donors were obtained from LifeSouth Community Blood Centers. The HC donors had recovered from COVID-19 and were positive for SARS-CoV2 antibodies at the time of blood donation. The donors had no prior clinically diagnosed autoimmune diseases. The samples were handled in a certified Biosafety Level 2+ (BSL2+) with Institutional Biosafety Committee-approved protocols.

#### National Institute of Dental and Craniofacial Research patients and protocols.

Patients were consented to NIH Central IRB-approved protocols (15-D-0051: *Characterization of Salivary Gland Disorders;* 20-D-0094: *Transmissibility and Viral Load of SARS-CoV-2 in Oral Secretions*) and evaluated at either the NIH SARS-CoV-2 Field Testing Facility (20-D-0094) or the NIH Clinical Center. NIH IRB Protocol: 15-D-0051 (NCT02327884) is a cross-sectional screening protocol to evaluate patients with a variety of disorders affecting the salivary complex and also healthy patients (i.e., HCs). All enrolled patients are evaluated comprehensively, including oral, sialometric, ophthalmologic, and rheumatologic evaluations, SG ultrasonography, blood work including rheumatologic investigations, and MSG biopsies. NIH IRB Protocol: 20-D-0094 (NCT04348240) was a short-term longitudinal study aimed at examining the potential transmissibility and viral load of SARS-CoV-2 in saliva when compared with nasal and nasopharyngeal secretions and for testing the effectiveness of masks to reduce speaking-related transmission (21). The general results of this study are reported in Huang et al. (21). After identifying SARS-CoV-2 in saliva, the protocol was amended to allow MSG biopsy in acute and convalescent COVID-19 patients (21).

Research and clinical records after initiation of the global COVID-19 pandemic were reviewed systematically by a rheumatology physician’s assistant. Patients were included in the histopathological analysis if they had recovered from COVID-19, had convalescent MSG biopsies, and were enrolled on NIH IRB Protocols: 15-D-0051 or 20-D-0094. Patients were excluded if they were evaluated as patients for the workup for SjD or non-SjD sicca symptoms. Comprehensive investigations as described above were completed on patients enrolled in our 15-D-0051 protocol. Still, due to constraints of NIH SARS-CoV-2 Field Testing Facility, these parameters could only be collected on some 20-D-0094 subjects. Clinical laboratory studies at NIH include standard blood work, assays for antinuclear antibodies, antibodies to extractable nuclear antigens (e.g., anti-SSA/SSB autoantibodies), and antibodies to pathogens to assess vaccination and exposure history purposes (e.g., antispike, antinucleocapsid). In 1 patient (Patient 2), serial MSG biopsies were collected; the first was taken 5 days after the first COVID-19 symptoms (reported previously as COV49 (21)), and the second was taken 6 months later (21).

MSG biopsies were interpreted by a board-certified anatomic pathologist for diagnostic purposes, and the histopathology was systematically reviewed by a board-certified oral and maxillofacial pathologist as previously described (48). Salivary gland inflammation and fibrosis were graded according to Greenspan et al. (49) and Tarpley et al. (50). For MSG with Greenspan grade 3 or 4 sialadenitis, an FS was calculated according to Daniels et al. (51). H&E, CD20, CD3, CD4, and CD8 were conducted by the Anatomic Pathology Laboratory of the National Cancer Institute. Slides were scanned at ′40 with a NanoZoomer S360 slide scanner (Hamamatsu Photonics), and digital photomicrographs at ×5 resolution were captured using NDP.view2 software (Hamamatsu Photonics).

### Statistics

Statistical analyses were performed using Prism 8 software (GraphPad). Where indicated, 2-way ANOVA, Welch’s *t* tests, or Mann-Whitney *U* tests were performed. In all cases, *P* values less than 0.05 were considered significant. For the antinuclear antibody staining, a Chi-squared test was performed.

### Study approval

Patients were consented to NIH Central IRB-approved protocols (15-D-0051: *Characterization of Salivary Gland Disorders; 20-D-0094: Transmissibility and Viral Load of SARS-CoV-2 in Oral Secretions*). Convalescent samples were collected under the University of Florida-approved protocol (IRB202000781). Participants gave informed consent to participate in the study before taking part. The University of Florida’s IACUC approved all protocols respective to breeding and the use of animals described herein. The experimental methods were carried out in accordance with the appropriate approvals and relevant guidelines.

### Data availability

The data sets generated and analyzed in the current study are fully available upon contact with the corresponding author. Values for all data points in graphs are reported in the Supporting Data Values file.

## Author contributions

For this study, AT performed the mouse infections. YS profiled the mice for SjD and generated and characterized the monoclonal antibodies. AV and LG performed the antinuclear antibody assays. MA, DEK, JOM, MB, EP, and BMW recruited patients and collected samples and data for analysis. ANB and KCH performed the PRNT assay. WFC, YS, and AV were involved with the histological examination. YS, AV, JAC, BMW, and CQN wrote the first draft of the manuscript. JAC, DAO, VR, SS, BMW, and CQN conceptualized the study, were involved in data analysis, and reviewed and edited the manuscript.

## Supplementary Material

Supplemental data

Supplemental table 3

Supporting data values

## Figures and Tables

**Table 1:**
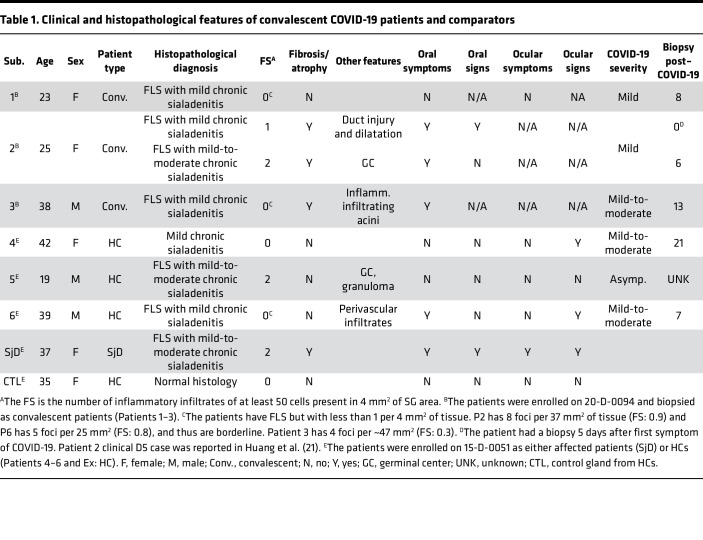
Clinical and histopathological features of convalescent COVID-19 patients and comparators

**Table 2:**
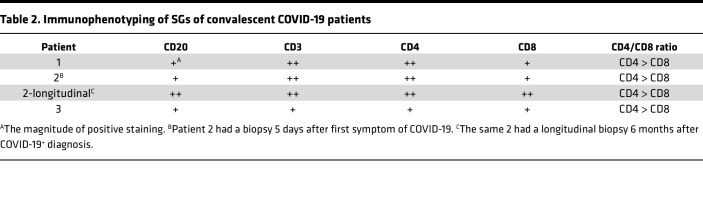
Immunophenotyping of SGs of convalescent COVID-19 patients

## Version 1 09/07/2023

In-Press Preview

## Version 2 12/22/2023

Electronic publication
