# Supplementary material for: Evidence of a Sjögren’s disease–like phenotype following COVID-19 in mice and humans
Source: JCI Insight. 2023 Dec 22;8(24):e166540. doi: 10.1172/jci.insight.166540 (PMC10807711; doi:10.1172/jci.insight.166540)
Supplement: Supplemental data [file jciinsight-8-166540-s174.pdf]

Evidence of a Sjögren's disease-like phenotype following COVID-19 in mice and human

Yiran Shen[1*], Alexandria Voigt[1*], Laura Goranova[1], Mehdi Abed[2], David E. Kleiner[3], Jose O. Maldonado[2,4], Margaret Beach[2], Eileen Pelayo[2], John A. Chiorini[4], William F. Craft[5], David A. Ostrov[6], Vijay Ramiya[7], Sukesh Sukumaran[8], Ashley N. Brown[9], Kaley C Hanrahan[9], Apichai Tuanyok[1], Blake M. Warner[2#], and Cuong Q. Nguyen[1,10,11#]

[1]Department of Infectious Diseases and Immunology, College of Veterinary Medicine, University of Florida, Gainesville, Florida, USA; [2]Salivary Disorder Unit, National Institute of Dental and Craniofacial Research, NIH, Bethesda, Maryland; [3]Laboratory of Pathology, Center for Cancer Research, National Cancer Institute, NIH, Bethesda, Maryland; [4]AAV Biology Section, National Institute of Dental and Craniofacial Research, NIH, Bethesda, Maryland, USA; [5]Department of Comparative, Diagnostic, and Population Medicine, College of Veterinary Medicine, University of Florida, Gainesville, Florida, USA; [6]Department of Pathology, Immunology & Laboratory Medicine, College of Medicine, University of Florida, Gainesville, Florida, USA; [7]LifeSouth Community Blood Centers, Gainesville Fl, [8]Valley Children's Hospital, Madera, California; [9]Institute for Therapeutic Innovation, Department of Medicine, University of Florida College of Medicine, Orlando, Fl; [10]Department of Oral Biology, College of Dentistry; [11]Center of Orphaned Autoimmune Diseases, University of Florida, Gainesville, Florida, USA.

*Authors contributed equally to the study

[#]Co-correspondence authors.

Address correspondence:

Cuong Q. Nguyen, PhD

Department of Infectious Diseases and Immunology

College of Veterinary Medicine, University of Florida

2015 SW 16th Ave, V3-152

Gainesville, Florida 32611-0880. USA

Telephone: 352-294-4180, Fax: 352-392-9704

nguyenc@ufl.edu

Blake M. Warner, DDS, PhD, MPH

Salivary Disorders Unit

National Institutes of Health

Building 10 Room 1A01

10 Center Drive

Bethesda, MD 20895

Telephone: 301-496-4486

blake.warner@nih.gov

METHODS

Animal models and LD_{50} determination:

The homozygous K18-hACE2 mice were bred at the University of Florida (Breeding Project ID #3276). Mating pairs of hemizygous K18-hACE2 mice were purchased from Jackson Laboratory (034860–B6.Cg-Tg(K18-ACE2)2Prlmn/J). F1 mice were anesthetized for ear punching for DNA extraction and subjected to zygosity analysis. The F1 zygosity (homozygosity) was confirmed via a SNP assay (The Jackson Laboratory's Protocol 38275: Sanger sequencing assay – Chr2_rs13476660-SEQ) by Transnetyx Inc. This assay detects a SNP between C57BL6/J and SJL/J (homozygous mutant), located approximately 4.7 kb from the transgene integration site. Once the homozygosity of the F1 mice was confirmed, mice between 8-10 weeks old were moved into micro-isolator cages, 5 mice per cage, under pathogen-free conditions for a challenge in the ABSL-3 laboratory. After being acclimated in the ABSL3 laboratory for three days, the mice were anesthetized with 87.5 mg/kg of ketamine and 12.5 mg/kg of xylazine of body weight by intraperitoneal (ip) injection. Once fully anesthetized, each group of mice was intranasally inoculated with 20 µL of SARS-CoV-2 inoculum drop-by-drop into both nostrils until fully inhaled. The mice received 860 PFU of SARS-CoV-2 WA1/2020 (BEI Resources, NIAID, NIH: SARS-Related Coronavirus 2, Isolate USA-WA1/2020, Recombinant Infectious Clone (icSARS-CoV-2-WT), NR-52281) (n=26). The naïve control mice (n=10) were inoculated with 20 µL of Dulbecco's Modified Eagle Medium (DMEM) supplemented with 2% fetal bovine serum (FBS). The mice were observed twice daily until the end of the study, 21 days. The mice were euthanized when moribund. The LD_{50} value was calculated using Probit analysis.

Making viral inoculum

One 150 cm^2 flasks (T-150) containing 75-80% confluent monolayer of Vero E6 (BEI Resources, NIAID, NIH: African Green Monkey Kidney Epithelial Cells (Vero E6) Expressing High Endogenous Angiotensin-Converting Enzyme 2, NR-53726) cells grown in Minimum Essential

Medium (MEM) Eagle supplemented with 10% heat-inactivated FBS was brought from the BSL-2 laboratory into a BSL3 laboratory two days before the experiment and incubated at 37°C with 5% CO_2. A frozen stock of 2 mL of SARS-CoV-2 WA1/2020 culture (BEI Resources, NIAID, NIH: SARS-Related Coronavirus 2, Isolate USA-WA1/2020, Recombinant Infectious Clone (icSARS-CoV-2-WT), NR-52281, Centers for Disease Control and Prevention)) was thawed at 37°C. After aspirating the culture medium from the T-150 flask, the thawed viral culture was inoculated onto the monolayer of the Vero E6 cells and was incubated for 1 hour. The viral inoculum was aspirated off and a freshly prepared 25 mL of MEM Eagle supplemented with 2% heat-inactivated FBS was added. The flask was incubated at the same growth condition as above. The monolayer was checked daily for signs of cytopathic effect. After 4 days of the infection, the viruses were harvested from the culture medium. The culture medium containing cells and the viruses was collected in a 50 mL conical tube. The tube was centrifuged at 4000 x g for 5 minutes to pellet the cells. The supernatant was collected and aliquoted into 2 mL vials before being stored in -80°C freezer until used. A solid double overlay plaque assay was used to quantify the viral particles as previously described(1).

Confirmation of infection by real-time PCR

15 mg of the lung tissues from SARS-CoV-2 infected K18-hACE2 mice were grinded using the Closed Tissue Grinder System (Fisher Scientific, Huston, TX). The single-cell suspension was obtained through a 70um cell strainer (Fisher Scientific, Huston, TX). The RNA was extracted through QIAamp® Viral RNA Mini Kit (QIAGEN, Germantown, MD), and 700 ng of extracted RNA was used for the Luna® SARS-CoV-2 RT-qPCR Multiplex Assay Kit (NEB, Ipswich, MA) to detect for nucleocapsid (N)1 and N2 genes of SARS-CoV-2 simultaneously. The samples were performed in duplicate. A Ct value of less than 40 was considered positive according to the manufacturer's guidelines. The copy number was calculated based on a manufacturer-provided positive control

Histological examination of salivary and lacrimal glands

Whole mouse heads were fixed in 10% phosphate-buffered formalin for at least 48 hours, then salivary and lacrimal glands were excised, embedded in paraffin, sectioned at a thickness of 5 µm, and stained for B Cells (BD, Franklin Lakes, NJ, 550286), T Cells (CD3: Santa Cruz Biotechnology, sc-1127; CD8: Santa Cruz Biotechnology, Dallas, TX, sc-20041), and macrophages (Genetex, Irvine, CA, GTX73723) as described previously(2). Stained samples were visualized using 400x magnification of Nikon Ti-E fluorescent microscope with an exposure of 200 milliseconds.

Human MSG were fixed immediately after biopsy in 10% neutral-buffered formalin for 24 hours, embedded in paraffin, sectioned at a thickness of 5 µm, and stained with hematoxylin and eosin. Histopathological assessment was performed and reviewed by a board-certified surgical pathologist (DK) and an oral and maxillofacial pathologist (BMW). When available, immunohistochemistry was performed to characterize immune infiltrates in the MSG using CD3 (clone 2GV6, 790-4341, Roche. Indianapolis, IN), CD20 (clone L26, 760-2531, Roche. Indianapolis, IN), CD4 (clone SP35, 790-4423, Roche. Indianapolis, IN), and CD8 (clone SP57, 790-4460, Roche. Indianapolis, IN). The intensity and pattern of the infiltrates were interpreted. All immunohistochemical studies used on anatomic pathology specimens were performed in the NCI Center for Cancer Research, Laboratory of Pathology, a laboratory approved by the College of American Pathologists Laboratory Accreditation Program. The included antibodies were clinically validated to the satisfaction of the College of American Pathologists' checklists and inspectors. Positive and negative tissue controls for each antibody are regularly checked and maintained in the Laboratory of Pathology.

Terminal deoxynucleotidyl transferase dUTP nick end labeling (TUNEL) and Caspase-3 immunofluorescence stains

Paraffin-embedded glands were deparaffinized via pressure cooking in Trilogy (Cell Marque, Rocklin, CA) for 10 minutes. Following a PBS-T wash, the sections were treated with proteinase K for 30 minutes. After a PBS-T wash, the positive control was incubated with 0.5 mg DNase I (Sigma Aldrich, Saint Louis, MO) for 10 minutes. Click-iT TUNEL Alexa Fluor Imaging Assays was performed as instructed by the manufacturer (ThermoFisher, Waltham, MA). Briefly, slides were blocked with a Tris-buffered solution, then incubated with terminal deoxynucleotidyl transferase (TdT) and Biotin-11-dUTP for 1 hour at 37 °C, before being stopped with sodium citrate butter. Slides were incubated with AF647 streptavidin and mounted with Vectashield DAPI-mounting medium. For caspase-3 detection, slides were blocked for an hour with donkey serum, followed by overnight incubation with 1:1,000 anti-caspase3 (Novus Biologicals, Littleton, CO, 9661T). Secondary antibody treatment consisted of 1:100 AF488 (ThermoFisher, Waltham, MA, A21206). Stained sections were mounted using a Vectashield DAPI-mounting medium. Stained samples were visualized at 10X magnification on Nikon Ti-E fluorescent microscope with an exposure of 200 milliseconds. Nikon NIS-Elements software was used to detect the threshold intensities utilizing the ROI function; threshold intensities were kept consistent throughout the experiment.

Detection of antinuclear antibodies

Antinuclear antibody detection in the sera of mice and humans and mAbs was performed per the manufacturer's instructions (Immuno Concepts, Sacramento, CA). Briefly, sera were diluted 1:40-1:320 in PBS and incubated on HEP-2 ANA slides for 30 minutes. For mAbs, undiluted samples were added where concentrations were all below 1.5 mg/ml. Goat anti-mouse IgG AF488 (Invitrogen, Waltham, MA, A11001) or manufacturer-provided anti-human IgG AF488 were added as secondary antibodies. Slides were sealed with Vectashield DAPI medium (Vector Laboratories, Burlingame, CA) and a glass coverslip was mounted. ANA staining pattern was

observed at 400x with a Nikon Ti-E fluorescent microscope with an exposure of 200 ms (Nikon, Tokyo, Japan).

Measurement of saliva flow

To measure stimulated flow rates of saliva, individual mice were weighed and given an intraperitoneal (ip) injection of 100 μl of a mixture containing isoproterenol (0.2 mg/1 ml of PBS) (Sigma-Aldrich, St. Louis, MO) and pilocarpine (0.05 mg/1 ml of PBS) (Sigma-Aldrich, St. Louis, MO). Saliva was collected for 10 min from the oral cavity of individual mice using a micropipette starting one minute after injection of the secretagogue. The volume of each saliva sample was measured and calculated in relation to the mouse's weight to produce saliva flow rate (SFR).

Immunohistochemical evaluation of SARS-CoV-2

Slides were deparaffinized and rehydrated, then antigens were retrieved using Antigen Unmasking Solution (Vector Laboratories, Burlingame, CA) per the manufacturer's instructions. Slides were blocked with goat sera in Avidin block (Vector Laboratories, Burlingame, CA), followed by mouse anti-SARS-CoV-2 Nucleocapsid protein (Bioss, Woburn, MA, BS-41408P) at 1:100 dilution with Biotin block (Vector Laboratories, Burlingame, CA) and incubated for 15 mins. After incubation with the secondary antibody (biotinylated goat anti-mouse IgG, Vector Laboratories, Burlingame, CA, BA-9200-1.5), ABC reagent (Vector Laboratories, Burlingame, CA) was added followed by DAB staining (Vector Laboratories, Burlingame, CA); counterstaining with hematoxylin was performed. Slides were scanned through Leica microscope at 40X. The results were analyzed on Aperio ImageScope viewing software (v12.4.3.5008, Leica).

Monoclonal antibody generation from convalescent COVID-19 patients

RBD (ACROBiosystems, Newark, DE) and S1 proteins (Novus Biologicals, Littleton, CO) were labeled with Dylight 488 and 650 (ThermoFisher Scientific, Waltham, MA), respectively per

the manufacturer's instructions. Convalescent patient PBMCs were stimulated as previously described(3) and cells were stained with DAPI, anti-CD20 PE (Biolegend, San Diego, CA, 302305), and 1:1,000 dilutions of the aforementioned SARS-CoV-2 proteins. Live, CD20+ B cells reactive against both RBD and S1 were sorted with the Sony SH800 Sorter (Minato City, Tokyo, Japan) into lysis buffer (Takara Bio Inc, Kusatsu, Shiga, Japan). Additionally, some heavy chains were recovered in a similar fashion by using SCAN(3) in which the capture slides were coated with anti-IgG (Invitrogen, Waltham, MA, 31130) and anti-Ig (SouthernBiotech, Birmingham, AL, 1010-01); captured antibodies were measured for reactivity to these proteins. Live B cells reactive against RBD were manually picked with a micromanipulator. The variable (V) heavy (H) (V_HDJ_H) and light (L) (V_LJ_L) chains of immunoglobulins (Igs) genes of individual cells were amplified using primer sets adapted from Rodda et al(4) and Tiller et al(5) for In-Fusion Cloning Kit (Takara Bio Inc, Kusatsu, Shiga, Japan). To express Igs, VH and VL were cloned into expression vectors pFUSEss-CHIg-hG1, pFUSE2ss-CLIg-hL2 and pFUSE2ss-CLIg-hK, respectively (Invivogen, San Diego, CA). The ligation products were transformed into competent Stellar™ Competent Cells (Takara Bio Inc, Kusatsu, Shiga, Japan,) and selected by Zeocin and Blasticidin resistance. Cloned plasmids were sequenced through GENEWIZ (Azenta, Chelmsford, MA) and aligned by NCBI IgBLAST to ensure sequences of chains were intact. To produce monoclonal antibodies (mAbs), FreeStyle™ CHO-S cells (ThermoFisher Scientific, Waltham, MA, R80007) were transfected with corresponding H/L expression vector DNA through FectoCHO® Expression System (PolyPlus, Vectura, France) as instructed by the manufacturer. On day 7 post-transfection, the cell culture was centrifuged and purified through EconoFit UNOsphere SUPrA on NGC™ Chromatography Systems according to the manufacturer (Bio-Rad, Hercules, CA). Protein concentration was quantified by Bradford protein assay (Quick Start™ Bradford 1x Dye Reagent, Bio-Rad, Hercules, CA) and the integrity of the Ig fractions with joined H/L chains was determined by Mini-PROTEAN TGX Stain-Free Precast Gels (Bio-Rad, Hercules, CA) by sodium dodecyl sulfate–polyacrylamide gel electrophoresis (SDS/PAGE).

Validation of neutralizing abilities of mAbs through competitive ELISA

The anti-SARS-CoV-2 neutralizing antibody titer serological assay kit (ACROBiosystems, Newark, DE) was used to measure the neutralizing capacity of mAbs according to the manufacturer. The samples were diluted to 2ug/mL, 1ug/mL and 0.5ug/ml. The samples were added to wells containing 0.3μg/mL of HRP-conjugated RBD that binds to the immobilized human ACE2 protein pre-coated on 96-well plates. The final absorbance was detected at 450 nm after adding substrate solution and stop solution. The results of the detected signal were normalized to the percentage of inhibition by comparing to the positive control, and a percentage of inhibition of 20% was considered to be inhibitory. CR3022 (produced under HHSN272201400008C and obtained through BEI Resources, NIAID, NIH: Monoclonal Anti-SARS Coronavirus Recombinant Human Antibody, Clone CR3022 (produced in HEK293 Cells), NR-52481) was used as a neutralizing antibody control.

Validation of neutralizing abilities of mAbs through Plaque Reduction Neutralization Test (PRNT)

PRNT assays were conducted on Vero E6 cells obtained from the American Type Culture Collection (ATCC; Manassas, VA, USA) with the USA-WA1/2020 strain of SARS-CoV-2. A description of cell maintenance and virus stock propagation is described elsewhere(6). Assays were conducted using BSL3 plus conditions employing protocols approved by the Institutional Biosafety Committee of the University of Florida (BIO5525 approved 11 March 2020). Antibodies were serially diluted 4-fold in MEM, yielding concentrations ranging from 0.0078 μg/ml to 2 μg/ml. A no-treatment control was also included for a total of 6 experimental conditions per antibody. The human IgG1 monoclonal antibody Adintrevimab (MedChemExpress, Monmouth Junction, NJ, USA) was employed as a positive control. Antibody dilutions were incubated with a total of 400 PFU of SARS-CoV-2 virus at 37°C for 1 h. Following the incubation period, 100 μl of each

antibody and virus mixture was inoculated onto confluent Vero E6 monolayers seeded in 6-well plates. Viral inocula were allowed adsorb for 1 h at 37°C, 5% CO2 before a primary overlay containing MEM supplemented with a final concentration of 0.5% agar (w/v) and 5% FBS (v/v) was added to each well. A secondary overlay comprised of MEM with a final concentration of 5% agar, 1% FBS, and 0.007% neutral red solution was overlaid two days later. Plaques were counted with the naked eye the following day.

Detection of SjD-associated autoantibodies in sera

Detection of human and mouse anti-SSA/Ro52, anti-SSA/Ro60, and anti-SSB/La was performed as previously described(7) Absorbance values of 450/630 nm were determined with a Tecan Infinite M200 Pro-spectrophotometric plate reader (TECAN, Mannedorf, Switzerland).

Detection of autoantibodies using INNO-LIA ANA Update Test strips

To analyze whether broad autoimmunity was preset in sera samples, INNO-LIA ANA Update Test strips (FujireBio Diagnostics, Tokyo, Japan) were utilized on human control (n=5 males, n=5 females) and COVID-19+ patient sera (n=10 males, n=10 females). In brief, sera were diluted 1:320 in sample diluent and 2 mL was loaded into each trough, with 2 mL Cut-off Control for the positive control strip. After incubating on rocker for one hour, LIA strips were washed and Conjugate Working Solution was added. After 30 minutes of rocking, LIA strips were washed and Substrate Solution was added. After 30 minutes of rocking, solution was aspirated and Stop Solution was added to LIA strips. After 15 minutes, LIA strips were dried and image was captured with a Gel Doc XR+ (BioRad, Hercules, CA). In the ImageLab 5.2.1 Software (BioRad, Hercules, CA), “lanes” (i.e. LIA strips) and “bands” were detected; every individual antigen band on every LIA strip was normalized against the Control LIA strip and correction was made for the altered sera concentration. Sera was considered positive if over the threshold (1± 2 standard deviations) utilizing the intensity of the internal controls provided on every LIA strip.

RESULTS

Table S1: Focal scores in salivary and lacrimal glands

	Mice (n)	Focal scores						Lymphocytic infiltration					
		Salivary glands			Lacrimal glands			Salivary glands			Lacrimal glands		
		Positive (n)	χ2	p-value	Positive (n)	χ2	p-value	Positive (n)	χ2	p-value	Positive (n)	χ2	p-value
**Control**	5	0	2.7	0.10247	0	13	0.00031	0	11	0.00091	0	24	0.00001
**Infected**	16	1			5			5			7		

Table S1: Incidence of exocrine-gland SjD symptoms in SARS-CoV-2 mice. The number of mice positive for a focal score (FS) in the salivary or lacrimal glands is provided, where an aggregate of ≥ 50 lymphocytes qualified as a single focus. Additionally, lymphocytic infiltration of B and/or T cells which did not qualify as a focus is reported for both the salivary and lacrimal glands. A McNemar's test was performed to compare the divergence of symptomology between the control and infected groups, reported as the χ^2, with p-values provided.

Table S2. SARS-CoV-2 reactive monoclonal antibodies.

mAb	V gene	D Gene	J gene	CDR3
**A10**	IGHV3-48*03	IGHD3-10*01	IGHJ6*02	ARDRVPNYYGSGSNYYGMDV
	IGLV7-43*01		IGLJ3*02	LLYYGGADWV
**B5**	IGHV1-69*08	IGHD1-26*01	IGHJ6*02	ARERPPKTDSGSYLYSYYYGMDV
	IGKV1-39*01	N/A	IGKJ4*01	QQSYSTPST
**C4**	IGHV1-8*01	IGHD3-22*01	IGHJ4*02	ATGRENYDTINYPLLTD
	IGLV2-14*03		IGLJ1*01	SSYTSSSTPYV
**C7**	IGHV3-23*04	IGHD3-22*01	IGHJ4*02	AKRLTDVVFISGDFDY
	IGLV2-23*02		IGLJ3*02	CSYAGSSTLV
**C9**	IGHV3-48*03	IGHD3-10*01	IGHJ6*02	ARDRVPNYYGSGSNYYGMDV
	IGLV3-19*01		IGLJ3*02	NSRDSSGRV
**D4**	GHV3-21*01	IGHD4-17*01	IGHJ4*02	ARDIYGDYYFDY
	IGLV2-14*03		IGLJ2*01, IGLJ3*01	QQYYTTPRT
**D5**	IGHV4-30-4*01	IGHD1-1*01, IGHD1-20*01, IGHD1-7*01	IGHJ4*02	ARAYWNPGHFDY
	IGKV3-15*01		IGKJ1*01	QQYNNWPRT
**S3**	IGHV4-30-4*01	IGHD3-22*01	IGHJ6*02	ARVVLVSSSYEGGMDV
	IGKV4-1*01		IGKJ1*01	QQYYTTPRT
**S8**	IGHV5-51*01	IGHD3-10*01, IGHD3-10*02	IGHJ6*02	ARNCYASGSYLSGMDV
	IGLV2-14*03		IGLJ1*01	SSYTSSSTPYV

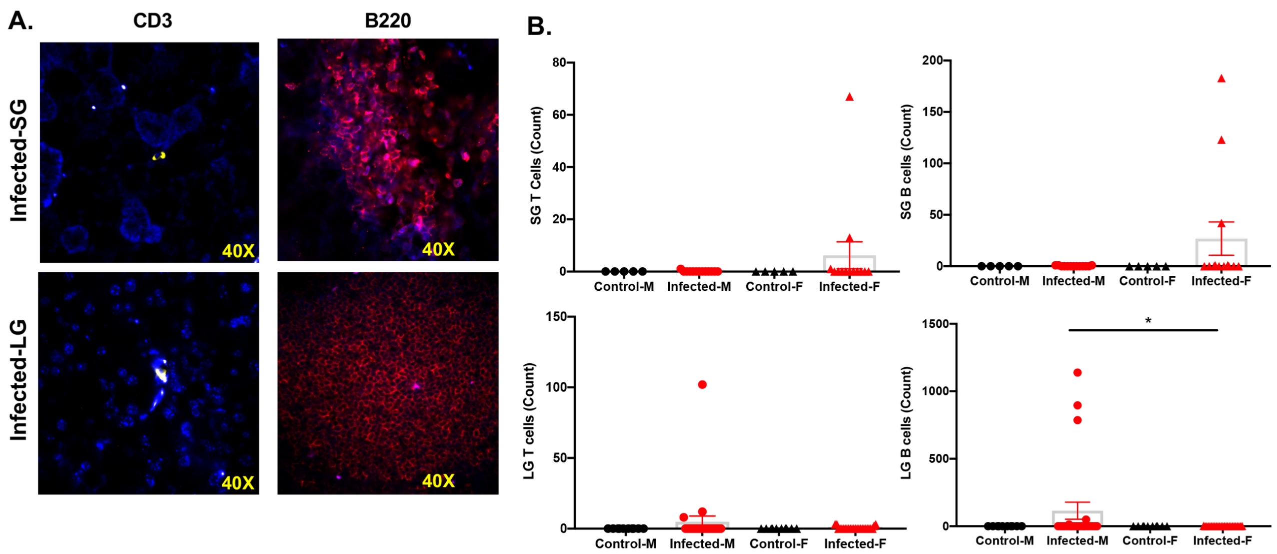

Figure S1: Frequencies of B and T lymphocytes in salivary and lacrimal glands of the infected mice. **A**) Identification of infiltrating cells in the salivary glands and lacrimal glands, where representative immunofluorescent staining of $CD3^+$ T cells and $B220^+$ B cells are displayed with blue DAPI nuclei staining at 40X magnification. **B**) Enumeration of B and T cells using. One-tailed Mann-Whitney t-tests were performed where * $p< 0.05$. SG: salivary glands, LG: lacrimal glands, M: Male, F: Female.

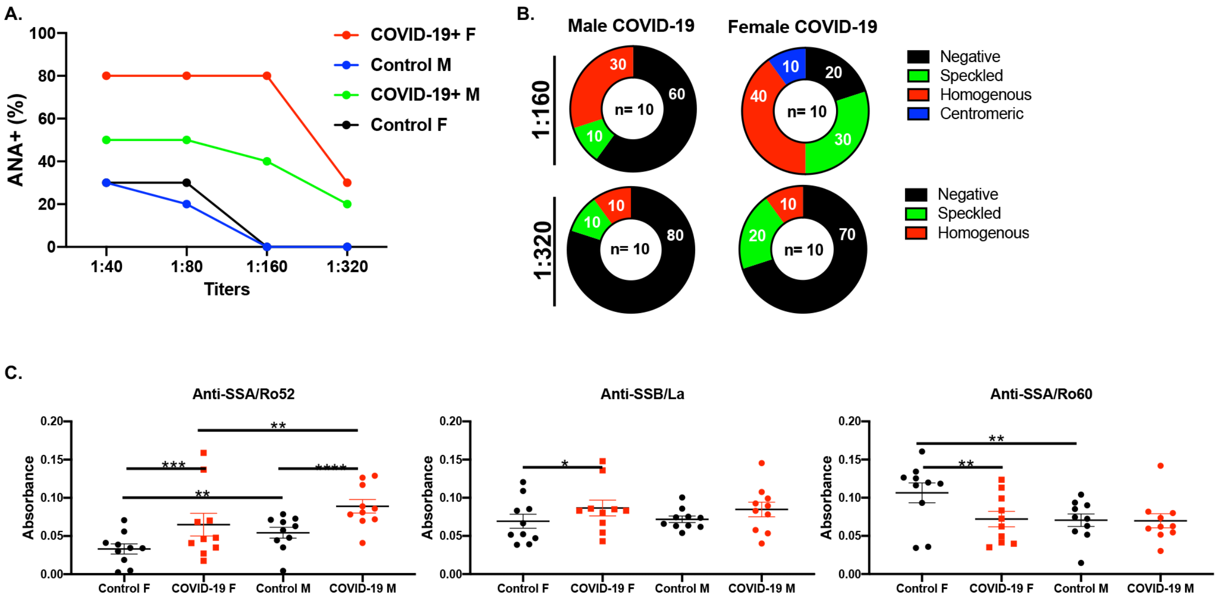

Figure S2: Sexual dimorphic autoantibody profiles of COVID-19 patients. A) The ANA frequencies of male and female patients and healthy controls were determined at various titers (n=10/group/sex). **B**) Staining patterns were determined at 1:160 and 1:320 titers where negative, speckled, homogenous, and centromeric staining patterns are indicated. Percentages of each staining are provided within the band and the total number of patients in the interior of circle. **C**) Anti-SSB/La, anti-SSA/Ro52, and anti-SSA/Ro60 levels are presented for males and females, control and COVID-19+ patients. Two-way ANOVA was performed to determine the significance of these results, where $^{**}p<0.01$, $^{***}p= 0.001$, and $^{****}p<0.0001$.

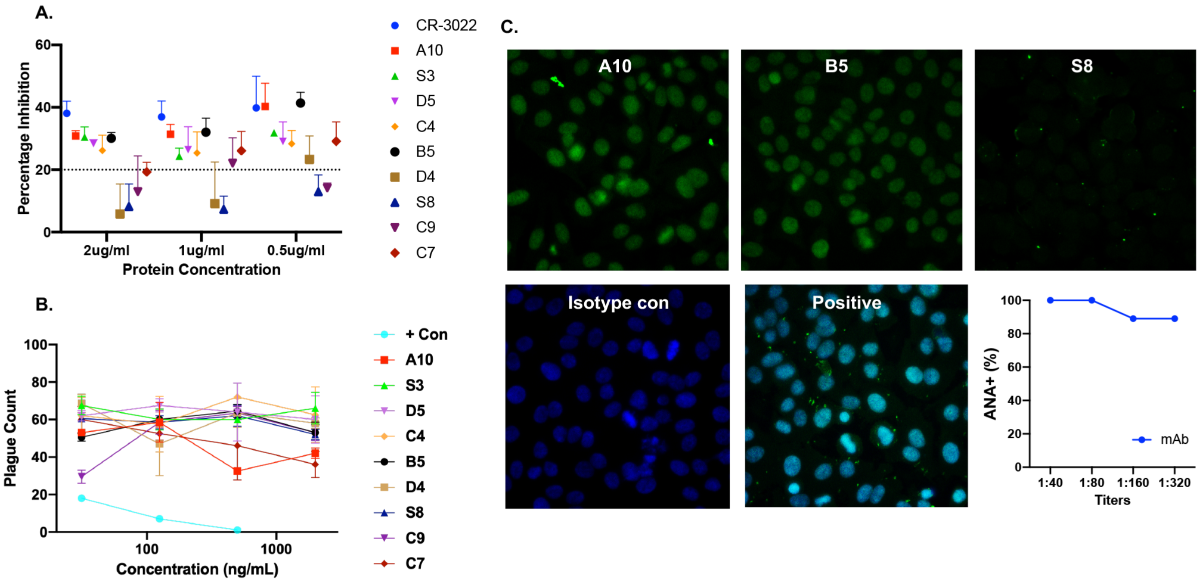

Figure S3: Monoclonal antibodies from recovered COVID-19 patients showed reactivity against self-antigens. A) Competitive ELISA demonstrated the comparable inhibition ability of SARS-CoV-2 RBD binding of nine mAbs under different concentrations, where CR-3022 (BEI Resources, NIAID, NIH: Monoclonal Anti-SARS Coronavirus Recombinant Human Antibody, Clone CR3022 (produced in HEK293 Cells), NR-52481) was a positive control. The dotted line indicates the threshold value of the inhibitory effect determined by the manufacturer. **B)** PRNT assays demonstrated the potential inhibitory ability of nine mAbs against SARS-CoV-2 infection at different concentrations. The human IgG1 monoclonal antibody Adintrevimab were used as positive controls. Viral inhibition was measured by plaque counts. The number of plaques is opposite to the inhibitory capacity. The dashed line indicates the mean counts of the plaque from all samples' negative controls as a reference. **C**) Nuclear antigen reactivity was examined using HEp2 cells. Seven of the nine S1/RBD-reactive mAbs produced a strong homogenous staining pattern at 1:40-1:320 titers. Representative images of three mAbs (A10, B5, and S8) are presented at the 1:320 titer with the blue DAPI turned off to better demonstrate the homogenous staining pattern. Isotype control and positive control were shown with DAPI and AF488.

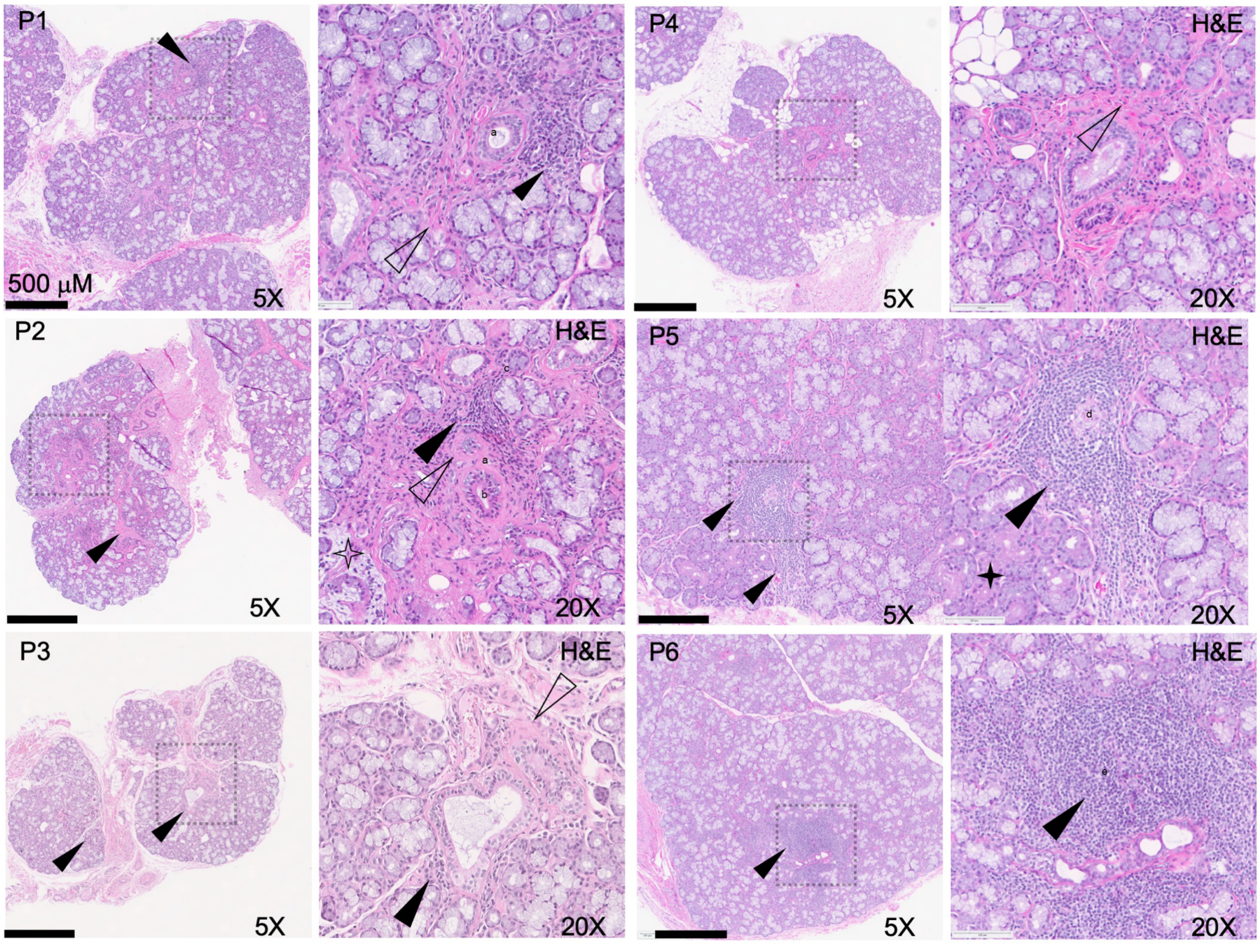

Figure S4: Hematoxylin and eosin (H&E) staining of salivary glands obtained from 6 patients recovered from COVID-19. Black arrows point to foci of inflammation and outlined arrows point to areas of fibrosis; open 4-pointed star shows an area of acinar necrosis adjacent to a focus of inflammation and prominent fibrosis; closed 4-pointed star shows oncocytosis of the acinar cells. Notably, P1, 2, 5, 6 all demonstrate FLS, although only 2/4 >1.0 foci/4mm^2. All patients show evidence of epithelial disorganization, injury of the ducts and acini, a: ductal injury, b: mucous inspissation, c: immune infiltration of the acini with injury, d: perivascular infiltrates, and e: granuloma.

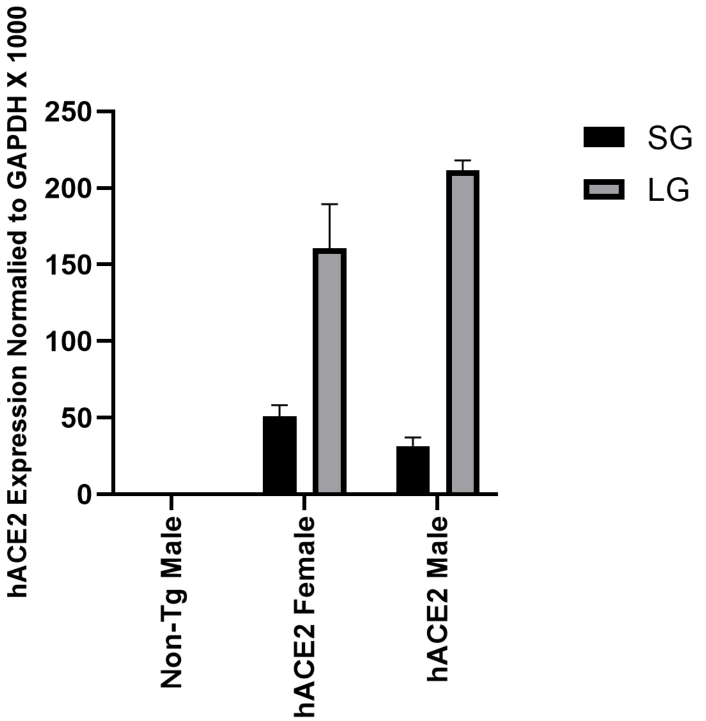

Figure S5. hACE2 expression levels in the salivary and lacrimal glands. Expression of human ACE2 (hACE2) mRNA in salivary glands (SG, black) and lacrimal glands (LG, grey) of control C57BL/6 mice and K18-hACE2 transgenic mice was determined by quantitative RT-PCR.

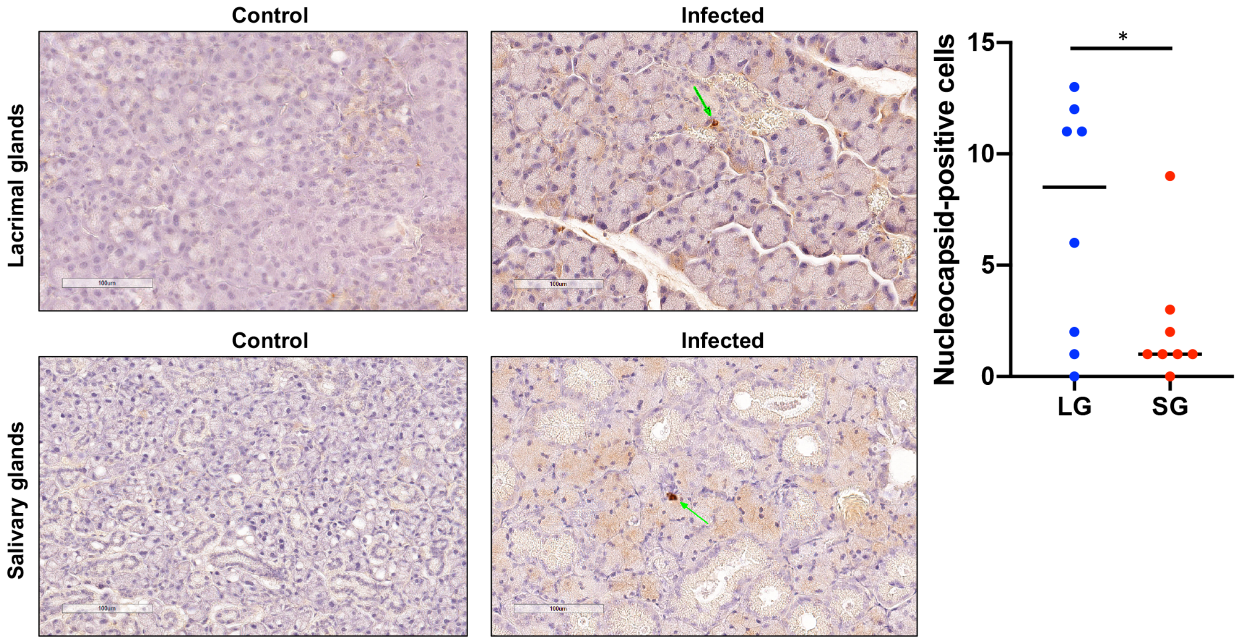

Figure S6: SARS-CoV-2 IHC staining in lacrimal glands (LG) and salivary glands (SG) from mice infected with SARS-CoV-2. **A**) Immunohistochemical analysis for SARS-CoV-2 nucleocapsid protein was performed with positive (arrow) in the LG and SG of infected mice. Control mice or uninfected mice showed no positivity for SARS-CoV-2 nucleocapsid. Original magnifications 20X; bar = 100 µm. **B)** Summary data of IHC staining for the number of DAB-positive cells in LGs and SGs of infected and control mice, with quantification of the data indicating the number of nucleocapsid protein positivity. A total of 8 infected mice (8 SGs and 16 LGs) were analyzed. One-tailed Mann-Whitney t-tests were performed where * $p < 0.05$.

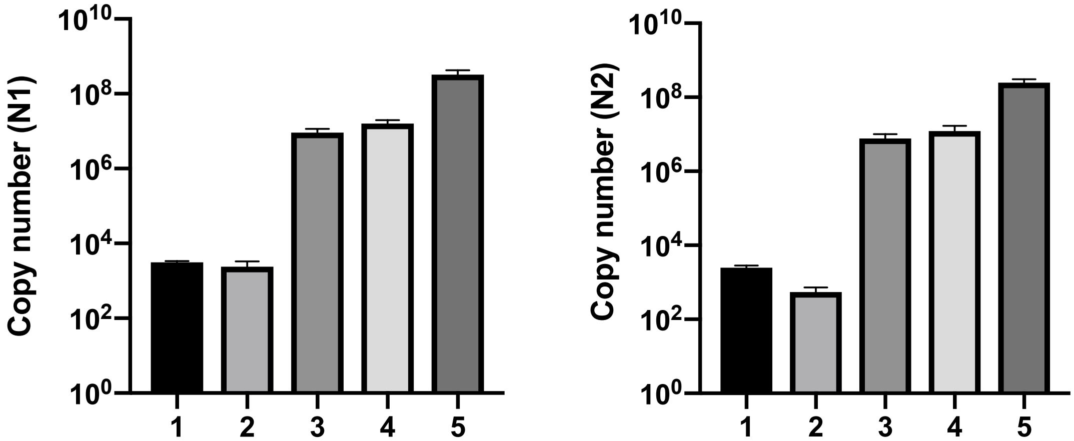

Figure S7: Determinination of viral loads in the lungs post infection. Mice received SARS-CoV-2 inoculum were randomly selected (n=5 with 3 females and 2 males). Viral RNAs were isolated from the lung tissues. Realtime PCR was performed to determine the copy number of the nucleocapsid (N)1 and N2 genes of SARS-CoV-2 as instructed by the manufacturer (NEB, Ipswich, MA). The samples were performed in duplicate. The copy number was calculated based on a manufacturer-provided positive control.

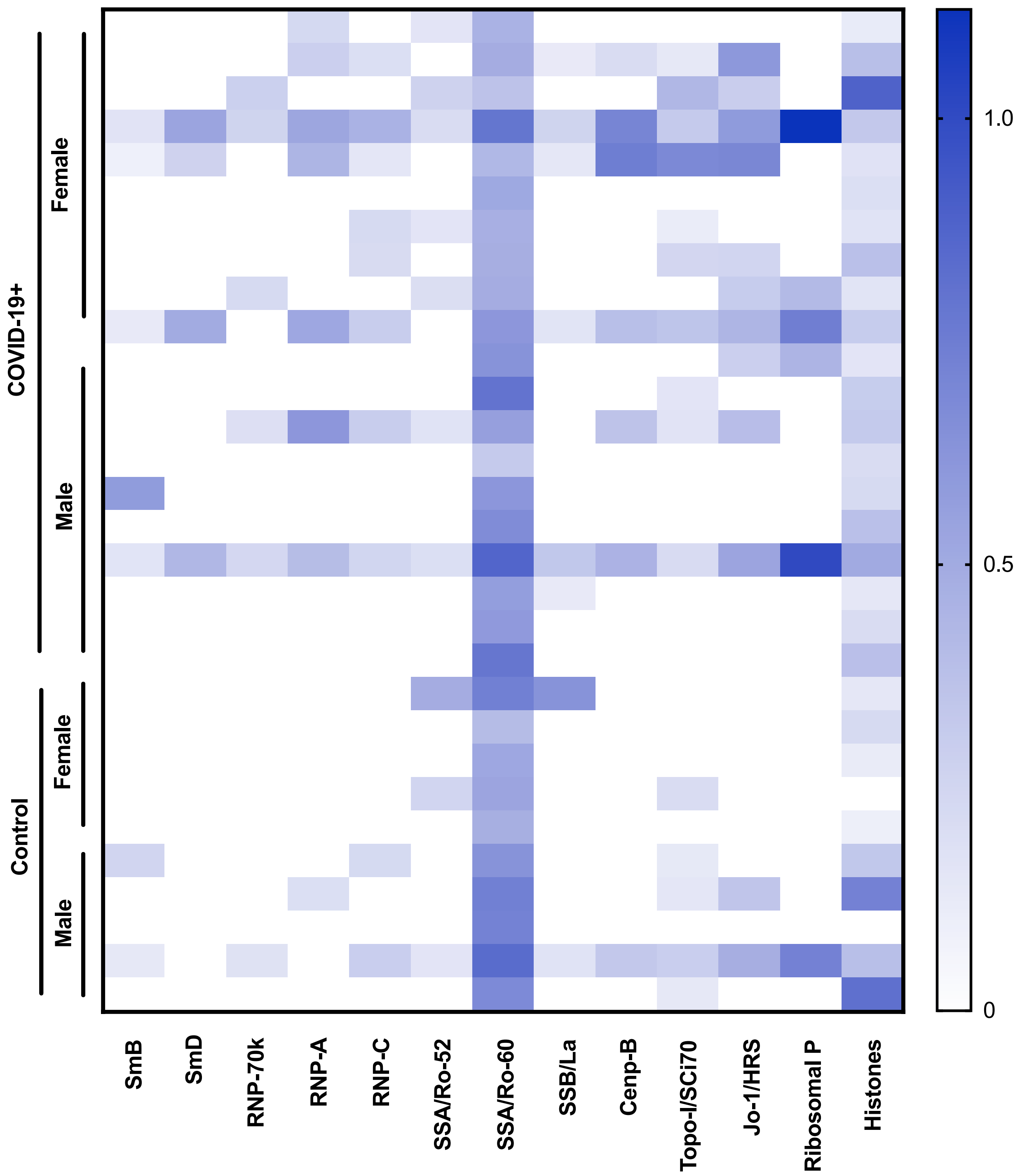

Figure S8: **Reactivity of COVID-19 patient sera against nuclear antigens.** Individual control males and females (n=5/group) and COVID-19+ males and females (n=10/group) are displayed via a representative heat map against 13 nuclear antigens. INNO-LIA strips were performed at a 1:320 dilution and normalized against a control stip. Darker blue indicates stronger reactivity

against the noted antigen, where significant binding occurs at a signal of 0.78 (1±2 standard deviations) or more.

Bibliography

1. Mendoza EJ, Manguiat K, Wood H, Drebot M. Two Detailed Plaque Assay Protocols for the Quantification of Infectious SARS-CoV-2. *Curr Protoc Microbiol* 2020;57(1):ecpmc105.
2. Witas R et al. Defective efferocytosis in a murine model of sjögren's syndrome is mediated by dysfunctional mer tyrosine kinase receptor. *Int. J. Mol. Sci.* 2021;22(18). doi:10.3390/ijms22189711
3. Esfandiary L et al. Single-cell antibody nanowells: a novel technology in detecting anti-SSA/Ro60- and anti-SSB/La autoantibody-producing cells in peripheral blood of rheumatic disease patients. *Arthritis Res. Ther.* 2016;18(1):107.
4. Rodda LB et al. Functional SARS-CoV-2-Specific Immune Memory Persists after Mild COVID-19. *Cell* 2021;184(1):169–183.e17.
5. Tiller T, Busse CE, Wardemann H. Cloning and expression of murine Ig genes from single B cells. *J. Immunol. Methods* 2009;350(1-2):183–193.
6. Brown AN, Strobel G, Hanrahan KC, Sears J. Antiviral Activity of the PropylamylatinTM Formula against the Novel Coronavirus SARS-CoV-2 In Vitro Using Direct Injection and Gas Assays in Virus Suspensions. *Viruses* 2021;13(3). doi:10.3390/v13030415
7. Gupta S, Li D, Ostrov DA, Nguyen CQ. Blocking IAg7 class II major histocompatibility complex by drug-like small molecules alleviated Sjögren's syndrome in NOD mice. *Life Sci.* 2022;288:120182.
